# TREM2 facilitates gastric cancer progression and immune evasion via inhibiting TRIM21-mediated STAT1 degradation in tumor-associated macrophages

**DOI:** 10.1038/s41419-025-08198-4

**Published:** 2025-11-18

**Authors:** Zihao Zhang, Kuan Yu, Yifan Cao, Peiyi Xie, Leihao Wang, Zhenbin Shen, Jing Qin

**Affiliations:** 1 Department of General Surgery, Zhongshan Hospital, Fudan University, Shanghai, China; 2 Gastric Cancer Center, Zhongshan Hospital, Fudan University, Shanghai, China; 3 Department of Liver Surgery and Transplantation, Liver Cancer Institute, Zhongshan Hospital, Fudan University, Key Laboratory of Carcinogenesis and Cancer Invasion, Ministry of Education, Shanghai, China

**Keywords:** Cancer microenvironment, Immune evasion

## Abstract

Tumor-associated macrophages (TAMs) play a crucial role in fostering an immunosuppressive tumor microenvironment, promoting cancer progression, contributing to immune evasion and resistance to immunotherapy. However, the mechanisms by which TAMs exert these effects in gastric cancer (GC) remain unclear. Human TAMs were isolated from GC patients through magnetic sorting for RNA sequencing. THP-1 cell line and mice bone marrow-derived macrophages (BMDMs) induced TAMs were applied for functional assays. Two in-house tumor microarrays were utilized for validation: the Zhongshan Cohort, comprising 135 patients, and the Zhongshan Flow Cytometry (ZSFC) Cohort, which included 60 patients. In this study, we identified a significant accumulation of TREM2^+^ TAMs in GC tissues, correlating with poor prognosis. Functional assays revealed that targeting TREM2^+^ TAMs suppressed GC progression both in vitro and in vivo. Mechanistically, TREM2 stabilized signal transducer and activator of transcription 1 (STAT1) by preventing its ubiquitination mediated by Tripartite Motif Containing 21 (TRIM21), while simultaneously promoting STAT1 phosphorylation via spleen-associated tyrosine kinase (SYK), leading to upregulation of CCL8 and PD-L1, fostering an immunosuppressive tumor microenvironment. Furthermore, depletion of TREM2^+^ TAMs significantly enhanced the efficacy of anti-PD-L1 immunotherapy in GC allograft models. Collectively, our findings establish TREM2^+^ TAMs as key drivers of GC progression and immune evasion. Targeting TREM2^+^ TAMs represents a promising therapeutic strategy to overcome resistance to anti-PD-L1 therapy and reshape the tumor immune microenvironment.

## Introduction

Gastric adenocarcinomas are the fifth most frequent malignancy and the fourth leading cause of cancer mortality globally. It has a 5-year relative survival rate of only 35.7% [1]. Despite multimodal neoadjuvant and adjuvant therapy, up to 50% of these individuals will have disease recurrence [2].

The booming immunotherapy of recent years endows cancer patients with better prognosis and clinical outcomes [3]. Gastric cancers (GC) with high tumor mutational burden (TMB), high microsatellite instability (MSI-H), EBV-related showed remarkable responses to immunotherapy [4]. However, the above-mentioned GC only accounts for 20% of the total number of GC patients [5]. The study showed the heterogeneity of tumor microenvironment (TME) accounts for the immunotherapy resistance in GC patients [6]. Thus, there is still a compelling need to distinguish the good and bad compositions in TME to potentiate the immunotherapy efficacy.

The TME includes a variety of immune cells, cancer-associated fibroblasts, endothelial cells, pericytes, and various other tissue-resident cells [7]. The cellular composition and functional status of the TME vary greatly depending on the organ of tumorigenesis, the intrinsic characteristics of cancer cells, tumor stage, and patient genetic profile, and are regulated by interactions between different cells [8]. Macrophages are the most abundant immune cells in the TME, which greatly affect the homeostasis of the TME and the patient’s response to immunotherapy [9]. Generally, macrophages were polarized to the M1 phenotype to exert an anti-tumor effect. However, tumor cells reprogram macrophages into M2 phenotype or tumor-associated macrophages (TAMs), which conduct immunosuppression function to support tumor progression [10]. It is reported that high infiltration of TAM predicts poor OS in GC patients [11]. Firstly, TAMs promote tumor progression by secreting cytokines like C-X-C motif chemokine, C-C motif chemokine, etc., therefore driving tumor cell proliferation and migration [12, 13]. Moreover, TAMs drive T cell exhaustion by upregulating immune checkpoint ligands like PD-L1, B7-S1, galectin-9, V-domain Ig-containing suppressor of T cell activation (VISTA), thus hindering the efficacy of immune checkpoint blockade immunotherapy [14–17]. Therefore, either by depleting immune suppressive TAMs or reprograming TAMs into immune stimulatory phenotype could be a feasible way of treating GC [18].

In the present study, through bioinformatic analysis and RNA sequencing, triggering receptor expressed on myeloid cells 2(TREM2) was identified as highly expressed in TAMs of GC. TREM2 is a transmembrane receptor belonging to the immunoglobulin superfamily, and it is mainly expressed on myeloid cells [19]. Previous research has proved that high TREM2 level predicts poor prognosis in various cancers [20–22]. Moreover, it is a pivotal molecule that regulates immunotherapy efficacy, probably by triggering T cell exhaustion [23, 24]. However, the function of TREM2 in TAMs and the relationships of TREM2^+^ TAMs with GC development remain elusive.

The present study found that TREM2^+^ TAMs promote GC progression by secreting CCL8, a pro-tumoral cytokine, and inhibiting CD8^+^ T cell function by increasing PD-L1 expression. Our findings suggest that targeting TREM2 can not only slow tumor progression and reshape the tumor immune microenvironment (TIME), but it can also improve the effectiveness of anti-PD-L1 therapy.

## Results

### TREM2 is up-regulated explicitly in TAMs and predicts a poor prognosis in GC patients

**Fig. 1:**
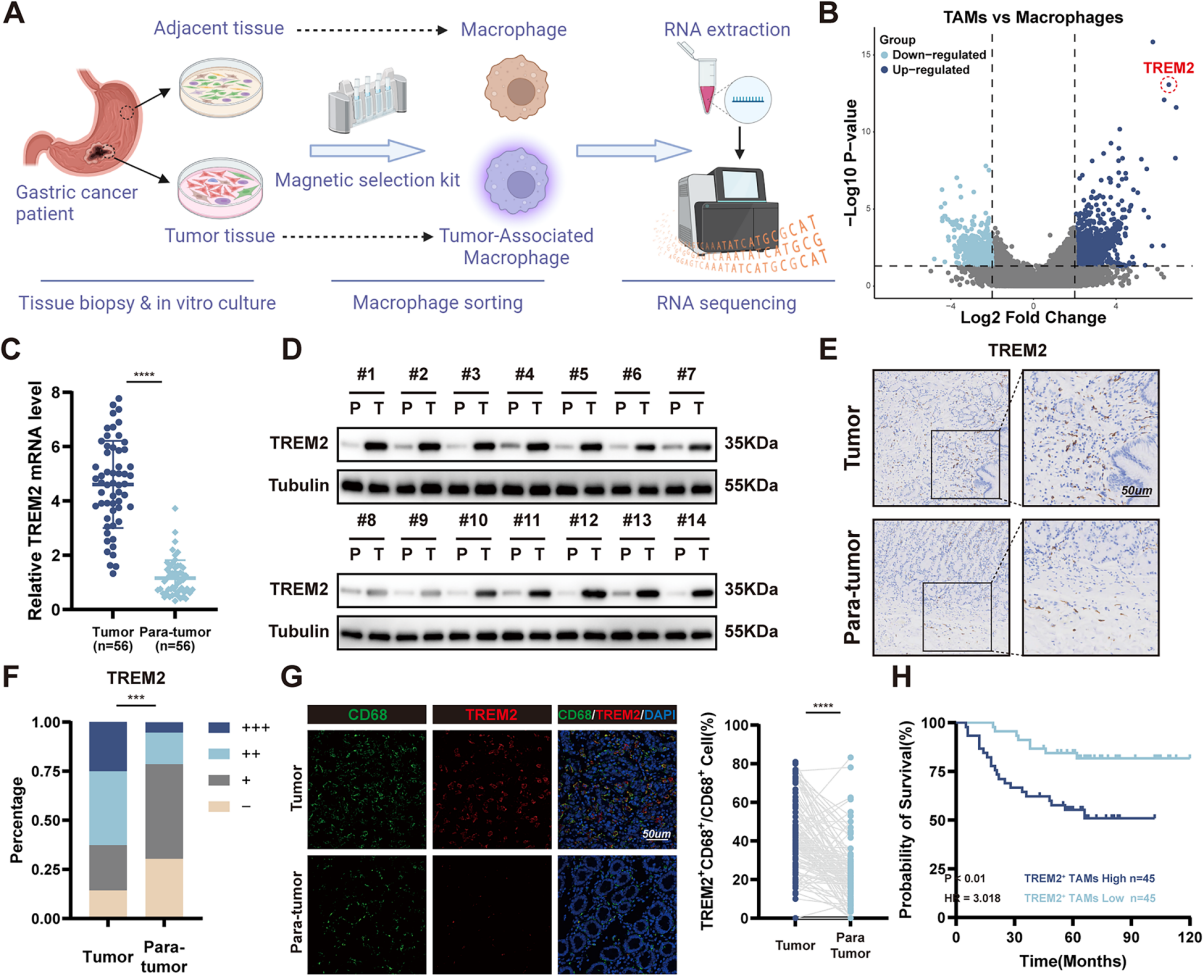
TREM2 is overexpressed in TAMs and is related to poor prognosis in GC patients. **A** Schematic diagram of sorting macrophages from tumor tissue and adjacent normal tissue. **B** Volcano plot of genes detected in RNA-seq from TAMs and normal macrophages. **C** mRNA expression of TREM2 in 56-paired GC tumor tissues and adjacent tissues. **D** Representative images of TREM2 expression in paired tumors and adjacent tissues from 14 GC patients using western blot. (**E**) Representative images IHC staining of TREM2 in 56-pairs of GC tissue and adjacent tissues. (**F**) Percentage of cases was analyzed by TREM2 expression. -, negative; +, low; ++, moderate; +++, strong. **G** Representative images and statistics data of the mIF staining of macrophage TREM2 in GC tissues and adjacent tissue in Zhongshan Cohort (*n* = 100). **H** OS curves based on TREM2 expression in Zhongshan Cohort (*n* = 100). Data are presented as mean ± SD. Statistical significance was analyzed using a Student’s *t*-test, Chi-square (X2) test, log-rank test. * *<*0.05, ** *<*0.01, *** *<*0.001, **** *<*0.0001.

To investigate the function of macrophages in GC development, TAMs and normal macrophages were isolated from tumor tissue and adjacent para-tumor tissue. First, surgical samples were digested into cell suspension. TAMs and normal macrophages for RNA sequencing were sorted through immunomagnetic beads and RNA-seq was performed to discover the difference between TAMs and macrophages (Fig. 1A). Differential expression gene (DEG) analysis showed TREM2 was one of the most significantly up-regulated genes in TAMs (Fig. 1B). Besides, public single cell dataset (GSE183904) proved TREM2 was mainly expressed in macrophages and upregulated in TAMs (Figure S1A-F). 56 pairs of GC and adjacent normal tissue were collected (Cohort 1). qPCR and WB proved TREM2 increased significantly in tumor tissues at transcription and protein level (Figs. 1C, D, S1G). Immunohistochemistry (IHC) demonstrated that, compared with adjacent normal tissue, TREM2 expression was enhanced in tumor tissues (Fig. 1E, F). Moreover, multiplex immunofluorescence (mIF) was applied to determine the amount of TREM2^+^ CD68^+^ cell infiltration in Zhongshan Cohort, then it categorized GC patients into high- or low-infiltration groups based on the mean TREM2^+^ cell density throughout the cohort. Kaplan-Meier analysis revealed that increased TREM2^+^ CD68^+^ cell infiltration was related to poor OS (Fig. 1G, H). Univariate, multivariate Cox regression analyses and ROC curves showed that higher infiltration of TREM2^+^ TAMs were independent predictors for postoperative OS (Fig. S1H, I). In summary, these findings indicate that TREM2 is predominantly expressed in GC TAMs, and its level is strongly correlated with GC prognosis.

### TREM2 stimulates M2 macrophage polarization and promotes GC cell proliferation and migration

**Fig. 2:**
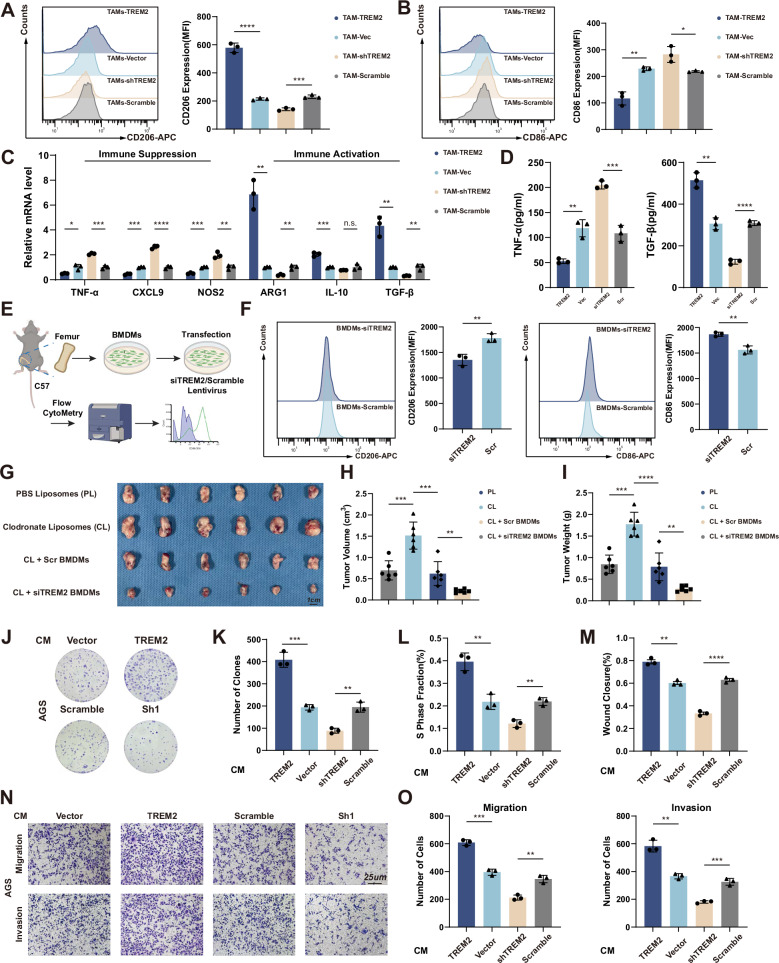
TREM2 promotes macrophages M2 polarization and GC cell proliferation and migration. **A**, **B** Flow cytometric analyses of CD206 and CD86 expression in TREM/shTREM2 and control TAMs. **C** Immune suppression and activation markers were detected by qPCR. **D** TNF-α and TGF-β were detected by ELISA in indicated cells. **E** Schematic diagram of extracting mice BMDMs. **F** Flow cytometric analyses of CD206 and CD86 expression in siTREM2 and control BMDMs. **G** Gross appearance of subcutaneous GC tumors from indicated groups. **H**, **I** The tumor weight and tumor volume of each group at the end point. **J**–**L** The Colony formation assay and the EdU assay were used to detect the effect of CM from TAMs on GC proliferation. **M**–**O** The wound healing and transwell assay were used to detect the effect of CM from TAMs on GC migration and invasion. Data are presented as mean ± SD. Statistical significance was analyzed using a Student’s *t*-test. * *<*0.05, ** *<*0.01, *** *<*0.001, **** *<*0.0001.

TAMs were induced and validated by increased expression of M2 marker CD206 (Fig. S2A, B). TREM2 were significantly expressed in THP-1 induced TAMs rather than gastric cancer cells lines (Fig. S2C, D). To determine the effects of TREM2^+^ TAMs on GC cells, the TREM2 overexpression TAMs (TREM2 TAMs) and TREM2 knockdown TAMs (shTREM2 TAMs) and corresponding control TAMs (Vector TAMs /Scramble TAMs) were constructed, respectively (Fig. S2E, G). Gain- and loss-of-function experiments revealed that TREM2 overexpression suppressed, whereas TREM2 knockdown promoted, the M1-like polarization of TAMs induced from THP-1 cells, which was evidenced by modulations in membrane expression of CD86 (M1 marker) and CD206 (M2 marker) (Fig. 2A, B) Besides, TREM2 upregulated immune suppression markers (*ARG1, TGF-β1, IL-10*) and TGF-β secretion, but downregulated immune activation makers (*NOS2, TNF-α, CXCL9*) and TNF-α secretion (Fig. 2C-D). Bone marrow-derived macrophages (BMDMs) were extracted from the femur of c57 mice transfected with the indicated adenovirus (Fig. 2E). Similarly, BMDMs transfected with siTREM2 adenovirus exhibited upregulated CD86 expression and downregulated CD206 expression compared to scramble BMDMs (Fig. 2F). Next, to verify the effect of TREM2^+^ TAMs on GC growth, we depleted macrophages in mice using clodronate liposomes and subsequently administered siTREM2 BMDMs or scramble BMDMs following subcutaneous tumor formation. The results showed that tumors were markedly smaller in mice receiving siTREM2-treated BMDMs compared with those receiving scramble BMDMs. (Fig. 2G-I). AGS and HGC-27 GC cell lines were used for co-culture experiments. Clone formation and EdU assay showed that TREM2 TAMs promoted, while shTREM2 TAMs inhibited GC cell proliferation (Figs. 2J–L, S3A, B). A co-culture transwell system was constructed to observe the effects of TAMs on GC cell migration and invasion. Transwell and wound healing assay showed that TREM2 TAMs promoted while shTREM2 TAMs inhibited GC cell migration and invasion (Figs. 2M–O, S3C, D).

### CCL8 secretion is essential for TREM2^+^ TAMs regulating GC cells

**Fig. 3:**
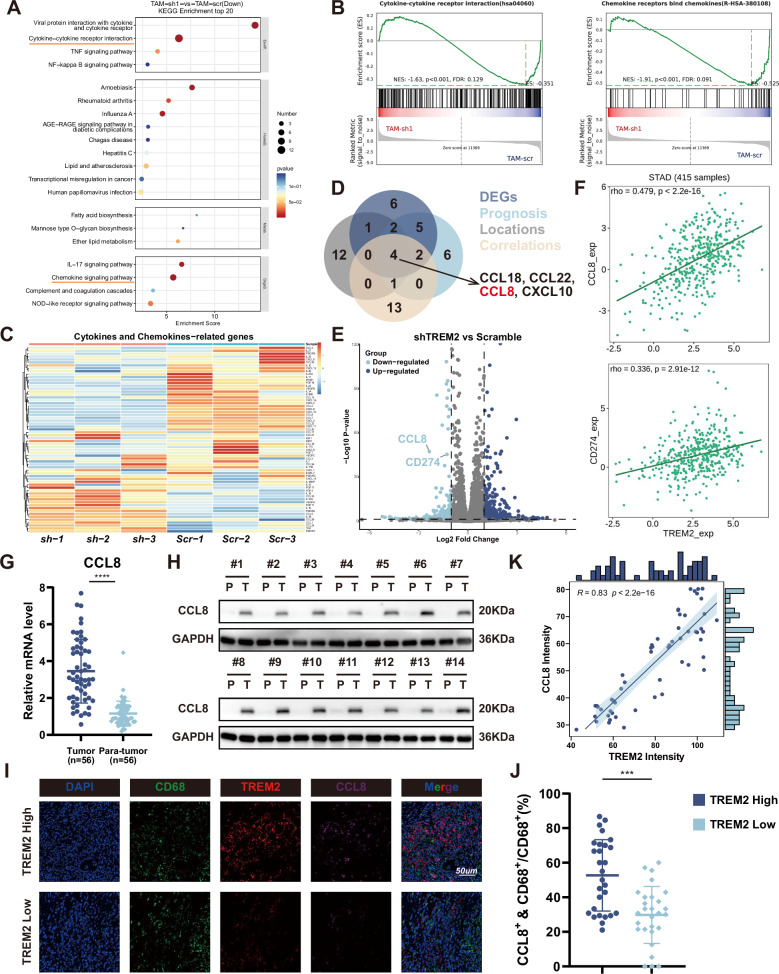
CCL8 secretion is essential for TREM2^+^ TAMs regulating GC cells. **A**, **B** KEGG and GSEA analysis between shTREM2 TAMs and Scramble TAMs. **C** Cytokines and chemokines-related genes regulated by TREM2 in RNA-seq data. **D** Cytokines and chemokines screening for the above genes depicted by venn diagram. (**E**) Volcano plots showing DEGs in the shTREM2 TAMs compared with scramble TAMs. |log2FC | > 1, *p*-value < 0.05. **F** Correlation analysis between TREM2 and CCL8 (upper panel) and PD-L1 (bottom panel) from TISIDB. **G** mRNA expression of CCL8 in 56-paired GC tumor tissues and adjacent tissues. **H** Representative images of CCL8 expression in paired tumors and adjacent tissues from 14 GC patients using western blot. **I** Representative images of the mIF staining of macrophage CCL8 and TREM2 in GC tumor tissues. **J** Percentage of CCL8^+^ macrophage based on TREM2 expression. **K** Correlation between macrophage CCL8 and TREM2 expression in GC tissues. Data are presented as mean ± SD. Statistical significance was analyzed using a Student’s *t*-test. *** *<*0.001, **** *<*0.0001.

RNA sequencing between shTREM2 TAMs and Scramble TAMs was performed to figure out the function of TREM2 in TAMs. Results revealed 269 downregulated and 209 upregulated genes with significance in shTREM2 TAMs compared with Scramble TAMs. Kyoto Encyclopedia of Genes and Genomes (KEGG) analysis revealed that the DEGs were mainly involved in cytokines and chemokines (Fig. 3A). In addition, gene set enrichment analysis (GSEA) showed that cytokines- and chemokines-related signaling pathways were significantly down-regulated in shTREM2 TAMs (Fig. 3B). Based on the cytokine and chemokine expression heatmap and restricted parameters, CCL8, primarily expressed by TAMs in GC tissue, was regarded as the potential candidate gene regulated by TREM2(Fig. 3C, D). CCL8 were upregulated in GC tissues and related to worse prognosis, as shown in TCGA and GEO datasets (Fig. S4A–E). Besides, the volcano plot showed that PD-L1 was downregulated after TREM2 knockdown in TAMs (Fig. 3E). The correlation analysis showed that the level of CCL8 and PD-L1 were positively correlated to TREM2 expression in the TCGA dataset (Fig. 3F). Then, transcription and protein level of CCL8 were detect in Cohort 1, the results proved that CCL8 was higher in tumor tissue than adjacent normal tissue (Fig. 3G, H). mIF confirmed that more CCL8^+^ TAMs were found in TREM2-High GC patients (Fig. 3I, J). Correlation analysis of patients in Zhongshan Cohort showed a strong positive relationship between the TREM2 and CCL8 (Fig. 3K).

### TREM2^+^ TAMs promote tumor progression in a CCL8-dependent manner

**Fig. 4:**
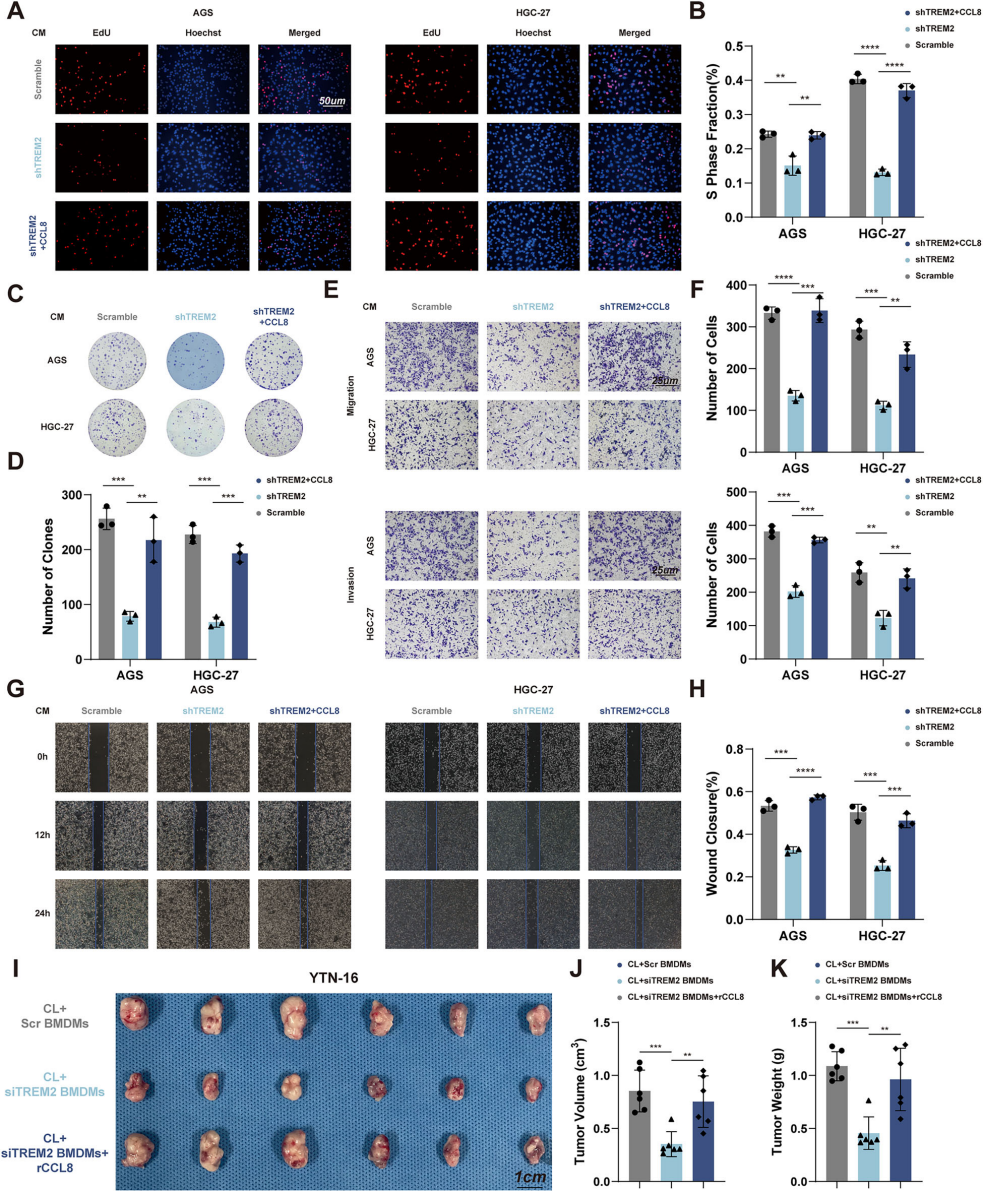
TREM2^+^ TAMs promote tumor progression in a CCL8-dependent manner. **A**–**D** EdU and Colony formation assays for cell proliferation of GC cells cultured by conditioned media from shTREM2 TAMs, with or without recombinant CCL8. **E**–**H** Transwell and wound healing assays were used to test cellular invasion and migration of GC cells cultured by conditioned media from shTREM2 TAMs, with or without recombinant CCL8. **I** Gross appearance of subcutaneous GC tumors from indicated groups. **J**, **K** The tumor weight and tumor volume of each group at the end point. Data are presented as mean ± SD. Statistical significance was analyzed using one-way ANOVA test. ** *<*0.01, *** *<*0.001, **** *<*0.0001.

We next validated whether CCL8 mediated the pro-tumor effect of TREM2^+^ TAMs. EdU and clone formation assay revealed that the addition of recombinant CCL8 to the CM from TAMs alleviated the TREM2 knockdown-mediated inhibition on GC cell proliferation (Fig. 4A–D). Co-culture transwell and wound healing assay showed that adding recombinant CCL8 to the CM from TAMs reversed the inhibitory effect of TREM2 knockdown on GC cell migration and invasion (Fig. 4E–H). Moreover, adding anti-CCL8 neutralizing antibody into the CM from TAMs suppressed the TREM2 overexpression-mediated promotion on GC cell proliferation and migration, as shown by EdU and wound healing assay (Fig. S5A, B). An in vivo experiment was performed to validate the function of CCL8 in tumor progression. The mice in the administration clodronate liposomes, recombinant CCL8 and siTREM2 BMDMs group bore larger tumor than those in the administration clodronate liposomes and siTREM2 BMDMs group (Fig. 4I–K). These results suggested that TREM2^+^ TAMs exerted pro-tumor effect through CCL8 secretion.

### TREM2 enhances CCL8 transcription in TAMs through eliciting phosphorylation of STAT1

**Fig. 5:**
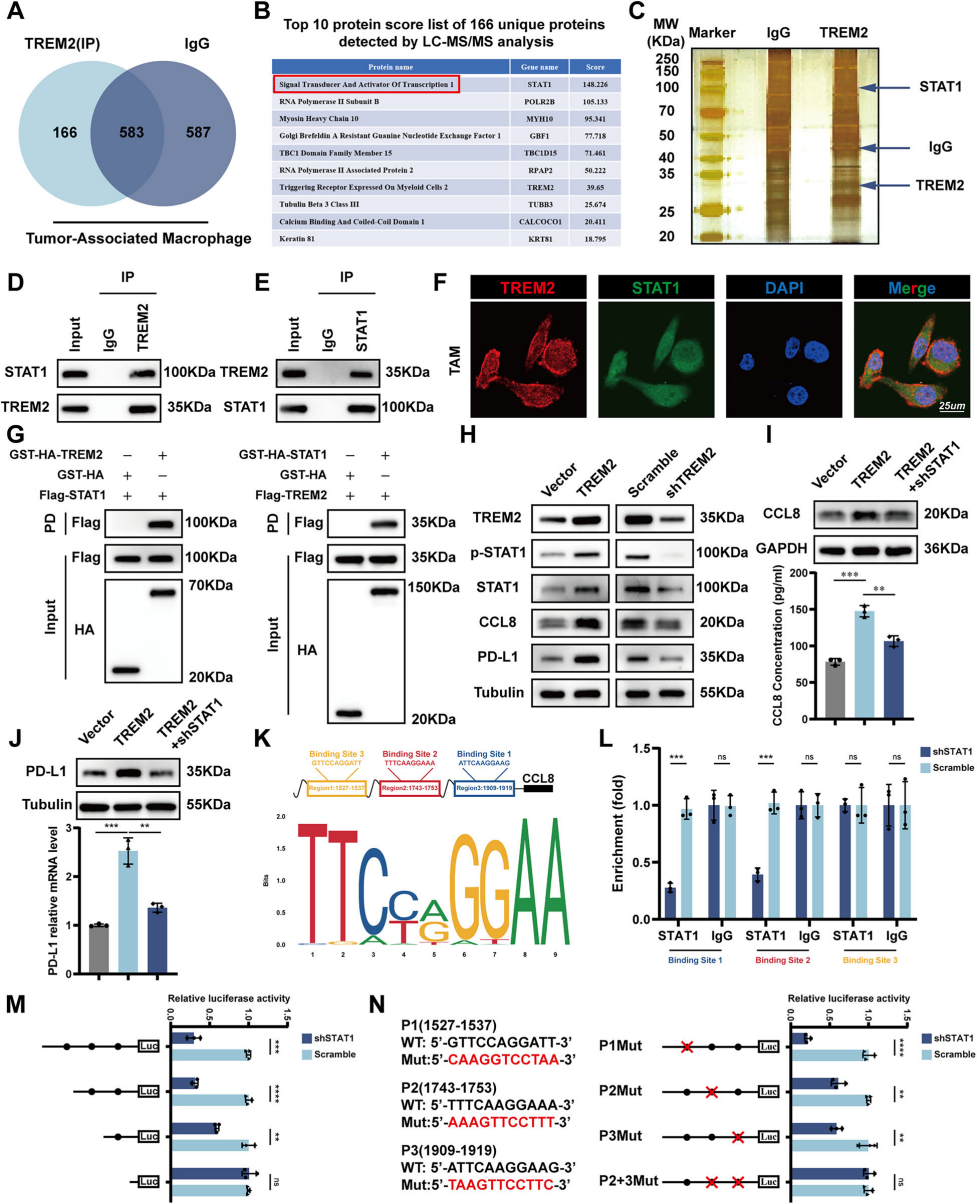
TREM2 regulates CCL8 and PD-L1 by promoting STAT1 phosphorylation. **A** Venn diagram presenting the overlapping potential unique TREM2-interacting proteins identified through LC-MS/MS analysis. **B** List of the top 10 potential unique proteins that interact with TREM2. **C** Silver staining of the gel. **D**, **E** Co-IP of endogenous TREM2 with STAT1 in TAMs. **F** Representative IF images of the colocalization of TREM2 and STAT1 protein. **G** GST pull-down assays were conducted to investigate the direct binding between TREM2 and STAT1. (**H**) Western blot of TREM2 and phosphorylated/non-phosphorylated STAT1, PD-L1, and CCL8 in the indicated TAMs. **I** The level of CCL8 was evaluated using western blot and ELISA in indicated groups. **J** The level of PD-L1 was evaluated using western blot and RT-qPCR in indicated groups. **K** Schematic of putative STAT1-binding sites in the CCL8 gene promoter region and the sequence logo and frequency matrix of STAT1. **L** ChIP PCR to investigate the binding of STAT1 to the CCL8 promoter sequences in indicated TAMs. **M** Relative luciferase activities of reporters containing full-length or fragments of the CCL8 promoter. **N** Relative luciferase activities of different reporters containing mutated sequences of the CCL8 promoter in the indicated TAMs. Data are presented as mean ± SD. Statistical significance was analyzed using a Student’s *t*-test and one-way ANOVA test. ** *<*0.01, *** *<*0.001, **** *<*0.0001, ns, not significant.

To elucidate the molecular mechanism by which TREM2 regulated CCL8 expression, liquid chromatography-tandem mass spectrometry (LC-MS) was applied to identify candidate proteins that might play critical roles in TREM2-induced CCL8 expression. A total of 167 potential unique TREM2-interacting proteins were identified. STAT1, which had the highest protein score, was regarded as the candidate protein that TREM2 interacts with (Figs. 5A–C and S6). Immunofluorescence (IF), co-immunoprecipitation (Co-IP) and GST pull-down assays together proved that TREM2 and STAT1 interacted in the cytoplasm of TAMs (Fig. 5D–G). Next, western blot showed that TREM2 stimulated the phosphorylation of STAT1 and upregulated the expression of CCL8 and PD-L1 (Fig. 5H). Moreover, WB, ELISA, and qPCR showed that STAT1 knockdown counteracted TREM2 induced upregulation of CCL8 and PD-L1 (Fig. 5I, J). As a transcription factor, p-STAT1 is capable of activating transcription of PD-L1; whether it could activate CCL8 transcription needs experimental validation. The online database Jaspar was then used to estimate probable p-STAT1 binding sites in the CCL8 promoter region, and the results showed 3 possible binding sites in the promoter region of CCL8 (Fig. 5K). Chromatin immunoprecipitation (ChIP) PCR showed that p-STAT1 was recruited exclusively to the promoter regions containing binding sites 1 and 2 (Fig. 5L). Luciferase reporter assays for binding site deletion or site-directed mutagenesis indicated that binding sites 1 and 2 in the CCL8 promoter induced p-STAT1-enhanced transcription activity (Fig. 5M, N).

### TREM2 binds to STAT1 to attenuate TRIM21-mediated STAT1 ubiquitination

**Fig. 6:**
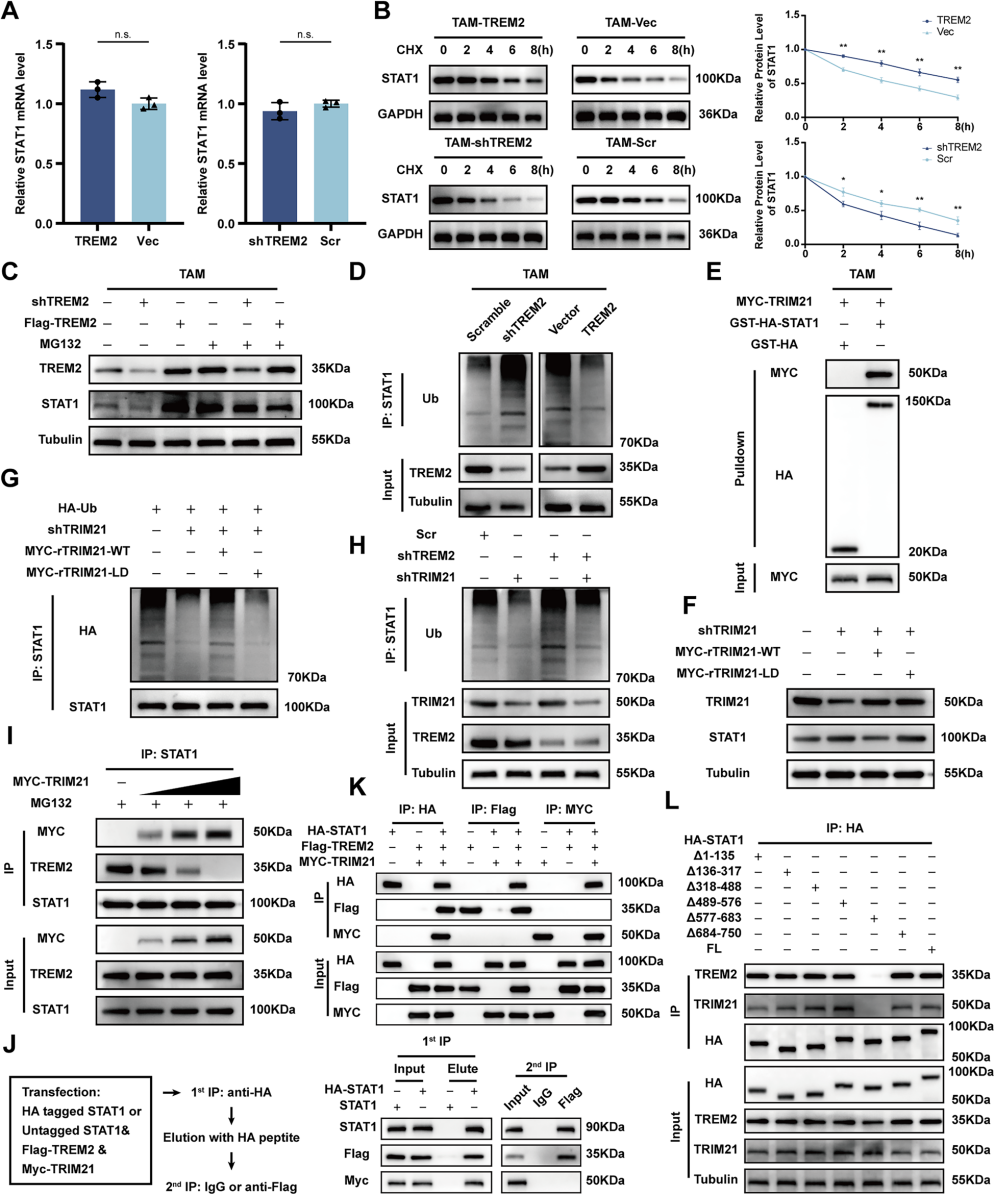
TREM2 competes with TRIM21 for STAT1 binding to attenuate TRIM21-mediated STAT1 ubiquitination. **A** qPCR analyses of the transcriptional levels of STAT1 in the indicated TAMs. **B** Western blot analysis of the effect of TREM2 on the half-life of STAT1 in TAMs treated with cycloheximide (CHX) (100 mg/mL) for the indicated time periods. **C** Western blot analysis of STAT1 in TREM2 overexpression and knockdown TAMs in the presence of MG132. **D** Western blot analysis of the ubiquitination level of STAT1 in TREM2 overexpression and knockdown TAMs. **E** GST pull-down assays were conducted to investigate the direct binding between TRIM21 and STAT1. **F**, **G** Western blot analysis of the protein and ubiquitination levels of STAT1 in TRIM21-knockdown cells transfected with wildtype (WT) or ligase-dead (LD) Flag-shRNA–resistant (r) TRIM21. **H** Western blot analysis of the effect of TRIM21 on ubiquitination level of STAT1 in TREM2 knockdown and scramble TAMs. **I** IP analysis of the interaction between TREM2, TRIM21, and STAT1 in HEK293T cells transfected with increasing doses of MYC-TRIM21 in the presence of MG132. **J** Left: Schematic showing the flow of sequential IP assays. Right: Co-IP analyses showed interactions of STAT1 with TREM2/TRIM21 but no binding of TREM2 to TRIM21. **K** Co-IP analysis of HEK293T cells co-transfected with Flag-TREM2, Myc-TRIM21, HA-STAT1. **L** Co-IP analysis of HEK293T cells transfected with either HA-tagged full-length STAT or STAT1 truncation mutants to assess domain-specific interactions.

To further investigate the molecular mechanism underlying the TREM2 enhancing the STAT1 pathway, mRNA level of STAT1 was detected in TREM2 overexpression and knockdown TAMs. However, TREM2 had no effect on the mRNA level of STAT1 (Fig. 6A). Therefore, it was reasonable to deduce that TREM2 regulated STAT1 via post-translational modification (PTM). A pulse-chase assay using cycloheximide revealed that STAT1 protein stability was reduced in shTREM2 TAMs, whereas TREM2 overexpression prolonged its half-life in TAMs (Fig. 6B). Moreover, to determine how TREM2 downregulates STAT1 stability, MG132 (a proteasome inhibitor) and CQ (an autophagy inhibitor) were applied and results showed that MG132 eliminated the downregulation of STAT1 in TREM2 knockdown TAMs, indicating TREM2 regulates the proteasome-dependent degradation of STAT1 (Figs. 6C, S7A, B). Furthermore, TREM2 overexpression in TAMs led to a decrease in STAT1 ubiquitination, whereas shTREM2 TAMs exhibited elevated ubiquitinated STAT1 levels in the presence of MG132 (Fig. 6D). Collectively, these findings suggest that TREM2 plays a crucial role in modulating the ubiquitin-mediated degradation of STAT1. Previous research proved tripartite motif containing 21 (TRIM21) was a E3 ubiquitin ligase responsible for the ubiquitination of STAT1 [25]. GST pull-down assays revealed that STAT1 bound directly to TRIM21 (Fig. 6E). According to *Zhou* et al., E3 ligase activity of TRIM21 is required for its ubiquitination function [26]. Therefore, to test this theory in TAMs, TAMs expressing shRNA-resistant wild type and ligase-dead TRIM21 were constructed, and results showed that TRIM21 promoted STAT1 degradation, however, the ligase-dead TRIM21 failed to perform its function (Fig. 6F, G). Notably, the elevated ubiquitination of STAT1 in shTREM2 TAMs was reversed by TRIM21 knockdown, suggesting that the effect of TREM2 on STAT1 stabilization is dependent on the expression of TRIM21 (Fig. 6H). Furthermore, the progressive increase in MYC-tagged TRIM21 weakened the interaction between TREM2 and STAT1 while simultaneously enhancing the binding between TRIM21 and STAT1. This dose-dependent effect suggests that TRIM21 competitively disrupts the STAT1-TREM2 association (Fig. 6I). Sequential immunoprecipitation assays demonstrated that STAT1 associated with TREM2 was unable to bind TRIM21. This finding was further confirmed through co-immunoprecipitation experiments, indicating that the interaction between TREM2 and STAT1 occurs in a mutually exclusive manner with TRIM21, suggesting competitive binding dynamics. (Fig. 6J, K). STAT1 is composed of six distinct domains: the N-terminal domain, coiled-coil domain, DNA-binding domain, linker domain, SH2 domain, and C-terminal transcriptional activation domain [27]. To identify the critical domains mediating STAT1 interactions with TREM2 and TRIM21, plasmids encoding the full-length STAT1 and six truncated mutants, each carrying a Flag tag, were generated and subjected to IP analysis. Our findings demonstrate that both TREM2 and TRIM21 interact with the SH2 domain (577–683 aa) of STAT1 (Fig. 6L). These results suggest that TREM2 and TRIM21 competitively associate with the SH2 domain of STAT1, with TREM2 acting to suppress STAT1 ubiquitination.

### TREM2 enhances STAT1 phosphorylation by potentiating the interaction of STAT1 and SYK

**Fig. 7:**
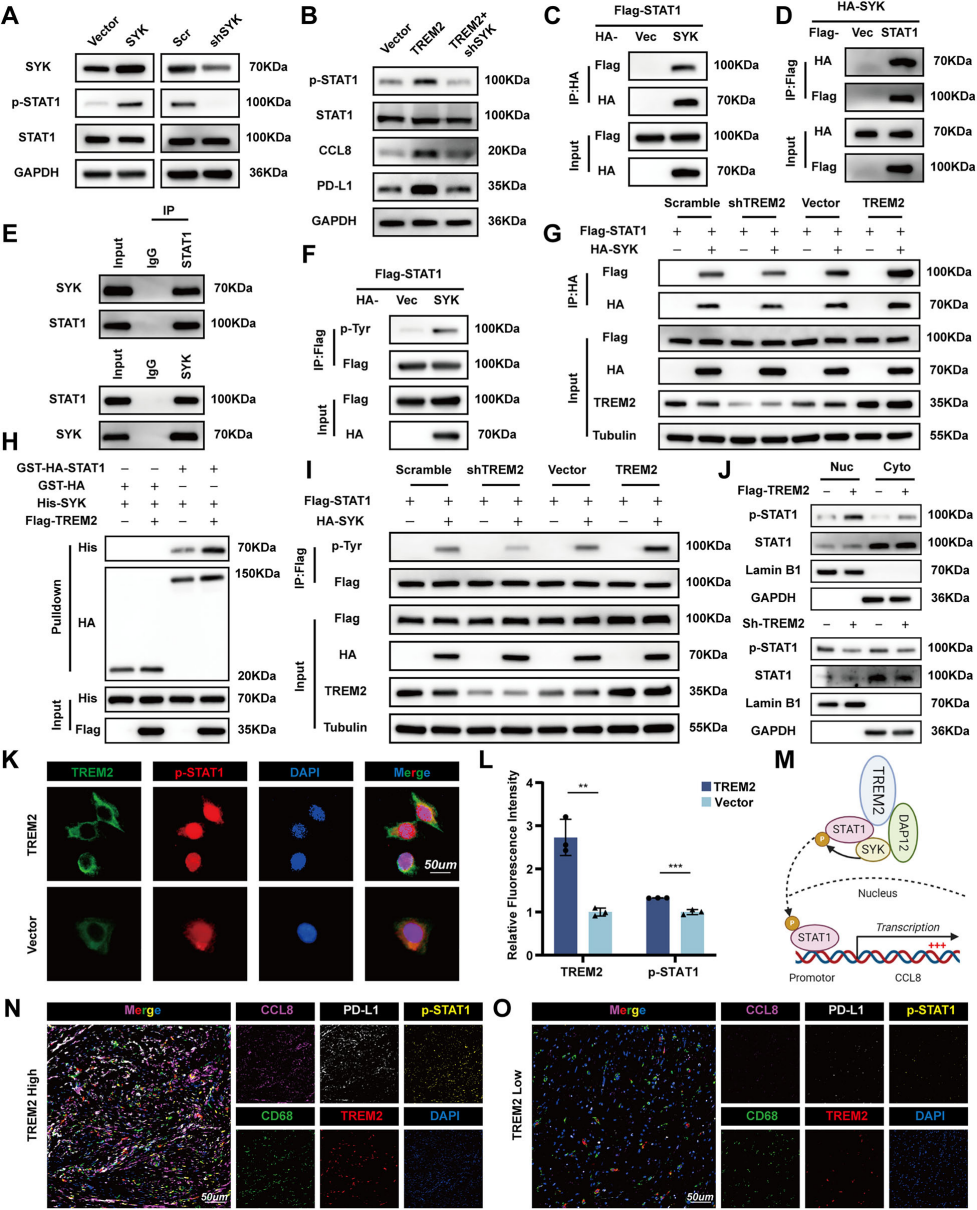
TREM2 interacts with STAT1 and promotes its phosphorylation through SYK. **A** Western blot of the protein and phosphorylation level of STAT1 in indicated TAMs. **B** Western blot of phosphorylated and non-phosphorylated STAT1, CCL8, and PD-L1 in the indicated TAMs. **C**, **D** Co-IP of exogenous SYK with STAT1 in HEK-293T. **E** Co-IP of endogenous SYK with STAT1 in TAMs. **F** Co-IP of exogenous phosphorylation of STAT1 in HEK-293T. **G** Co-IP of interaction between STAT1 and SYK in TREM2 overexpression and knockdown TAMs. **H** GST pull-down assays were conducted to investigate the direct binding between STAT1 and SYK with or without TREM2. **I** Co-IP of phosphorylation of STAT1 in TREM2 overexpression and knockdown TAMs. **J** Western blot of cytoplasmic and nuclear phosphorylated and non-phosphorylated STAT1 in TREM2 overexpression and knockdown TAMs. **K**, **L** IF images of the colocalization of TREM2 and p-STAT1 protein. **M** Schematic diagram showing TREM2 recruits STAT1 and promotes its phosphorylation through SYK, which, in turn, upregulates CCL8 transcription in TAMs. **N**, **O** Representative mIF images of fresh GC tumor sections. Data are presented as mean ± SD. Statistical significance was analyzed using a Student’s *t*-test. ** *<*0.01, *** *<*0.001.

TREM2, as a membrane receptor, receives stimulus from the environment and conducts signals to downstream molecules, which execute the biological function. SYK, binding to TREM2 through DAP12, activating PI3K, ERK, etc., is a key molecule in the TREM2 signaling pathway. Thus, we deduce that TREM2 promotes STAT1 phosphorylation through SYK. SYK overexpression (SYK TAMs), knockdown TAMs (shSYK TAMs), and corresponding control TAMs (Vector TAMs / Scramble TAMs) were constructed. Despite the level of SYK, the STAT1 remained unchanged; however, the phosphorylation of STAT1 was upregulated as SYK increased (Fig. 7A). Furthermore, SYK knockdown abolished the promotion on STAT1 phosphorylation as well as upregulation of CCL8 and PD-L1 induced by TREM2 overexpression (Fig. 7B). Exogenous and endogenous Co-IP showed SYK interacted with STAT1 and SYK promoted the phosphorylation of STAT1 (Fig. 7C–F). Co-IP in indicated cells proved TREM2 could promote the interaction between SYK and STAT1 (Fig. 7G). GST pull-down assays revealed that TREM2 served as a scaffold to promote the SYK-STAT1 direct interaction (Fig. 7H). Moreover, TREM2 enhanced the phosphorylation of STAT1 induced by SYK (Fig. 7I). WB and immunofluorescence showed TREM2 promoted p-STAT1 formation and nucleus translocation (Fig. 7J–M). To further verify the identified TREM2/p-STAT1/CCL8, and PD-L1 axis in clinical GC tissues, we examined the expression of TREM2, p-STAT1, CCL8 and PD-L1 by mIF in Zhongshan Cohort (Fig. 7N, O). A positive correlation between TREM2 and p-STAT1, CCL8, and PD-L1 was detected (Fig. S8A–C).

### Targeting TREM2 on TAMs potentiates the efficacy of anti-PD-L1 therapy and reverses the immunosuppression TME

**Fig. 8:**
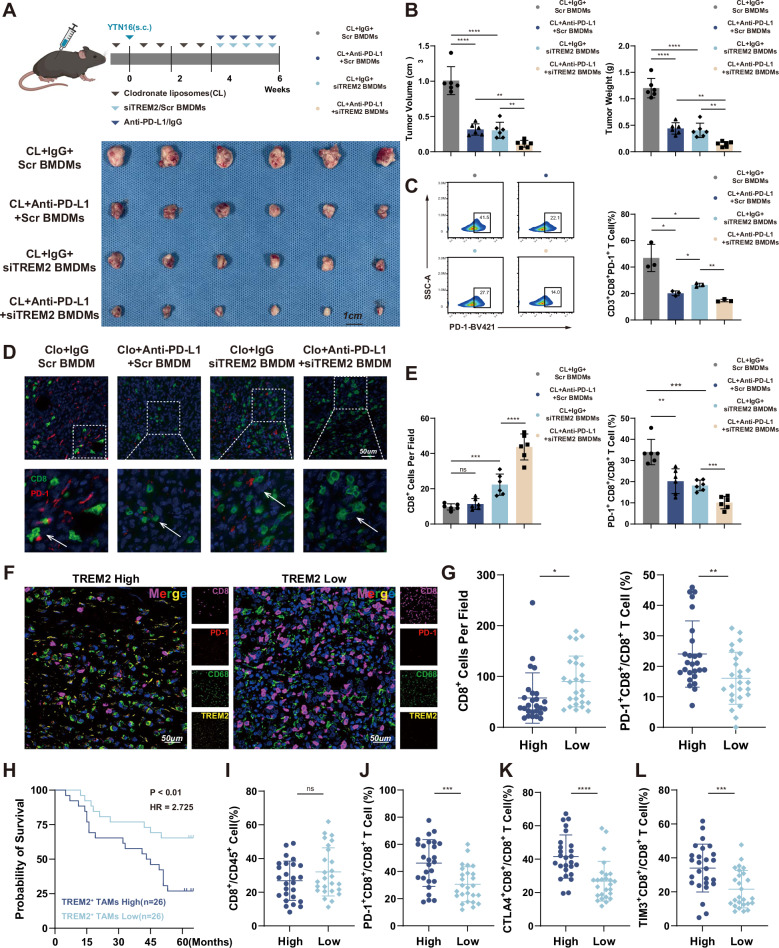
Targeting TREM2 on TAMs enhances anti-PD-L1 efficacy by down-regulating PD-1^+^CD8^+^ T cell. **A** Schematic showing the schedule for administration of the siTREM2 BMDMs and the anti-PD-L1 antibody to allograft tumor model mice (up panel), gross appearance of subcutaneous GC tumors from indicated groups (bottom panel). **B** The tumor weight and tumor volume of each group at the end point. **C** Flow cytometric analysis of the proportions of infiltrating CD3^+^ CD8^+^ PD-1^+^ T cells in tumors from allograft tumor model mice receiving the indicated treatments. **D** Representative mIF of the tumor-infiltrating PD1^+^CD8^+^ T cell in indicated mice group. **E** Number of CD8 + T cell in indicated mice group and proportion of PD-1^+^ CD8^+^ T cell in indicated mice group. **F** Representative mIF images of GC tumor sections in ZSFC cohort. **G** Number of CD8^+^ T cells and the proportion of PD1^+^CD8^+^ T cells in each case of ZSFC cohort. **H** OS curves based on TREM2 expression in patients with GC from ZSFC cohort. **I**–**L** Flow cytometric analysis of the proportions of CD8^+^ T cell, PD-1^+^CD8^+^ T cell, CTLA4^+^CD8 + T cell and TIM3^+^CD8^+^ T cell of patients from ZSFC cohort. Data are presented as mean ± SD. Statistical significance was analyzed using one-way ANOVA test. * *<*0.05, ** *<*0.01, *** *<*0.001, **** *<*0.0001, ns, not significant.

As TREM2 upregulated PD-L1 on TAMs, whether targeting TREM2 on TAMs sensitized anti-PD-L1 treatment was tested in c57 mice. Mice bearing subcutaneous YTN-16 GC tumors were administrated with the siTREM2 BMDMs and/or an anti-PD-L1 antibody (Fig. 8A). More substantial inhibition of tumor development was observed in mice treated with the combination therapy than in those given with either the control BMDMs or monotherapy at the endpoint (Fig. 8B). Flow cytometric analysis showed that less PD-1^+^CD8^+^ T cells were infiltrated into tumors in mice treated with combined therapy (Fig. 8C). Furthermore, as demonstrated by the mIF, mice treated with combination therapy recruited more CD8^+^ T cells and decreased the proportion of PD-1^+^CD8^+^ T cells in tumor tissues (Fig. 8D, E). Moreover, in ZSFC cohort, high infiltration of TREM2^+^ TAMs led to total CD8^+^ T cell depletion while a higher proportion of PD-1^+^ CD8^+^ T cells (Fig. 8F, G). Besides, ZSFC cohort showed high TREM2 predicts a worse prognosis for GC patients (Fig. 8H). Corresponding flow cytometric data validated that higher TREM2^+^ TAMs infiltration led to a higher proportion of PD1^+^, CTLA4^+^, and TIM3^+^ CD8^+^ T cells (Fig. 8I-L). These results indicate that targeting TREM2 on TAMs can enhance the sensitivity to anti–PD-L1 therapy by increasing CD8⁺ T-cell infiltration and reducing the proportion of exhausted CD8⁺ T cells.

## Discussion

Gastric cancer (GC) has a distinct and diverse tumor microenvironment (TME), which accounts for the poor response to immune checkpoint blockade (ICB) [28, 29]. Tumor-associated macrophages (TAMs), the most abundant immune cells, play a vital role in the TME and are involved in multiple key biological processes like angiogenesis, extracellular matrix remodeling, cancer cell proliferation, metastasis, and resistance to checkpoint blockade immunotherapy [9]. Here, we found that TREM2^+^ TAMs were accumulated in GC, promoting PD-L1 expression and CCL8 secretion through STAT1-enhanced transcription, leading to cancer progression and CD8^+^ T cell exhaustion. Clinically, our data revealed that targeting the TREM2^+^ TAMs is a promising strategy for potentiating anti-PD-L1 therapy.

The TREM2 receptor is a transmembrane protein that consists of an extracellular region, a membrane-spanning region, and an intracellular region [30]. Accumulating evidence has revealed the immunosuppression role of TREM2^+^ TAMs in several cancers [31, 32]. While, in pancreatic ductal adenocarcinoma, TREM2 deficiency promotes tumor progression through the expansion of proinflammatory macrophages and IL-1β–driven pathogenic inflammation [33]. Moreover, the TREM2^+^ TAMs enrichment was a main trait in hepatocellular carcinoma patients who were resistance to atezolizumab + bevacizumab therapy [34]. However, the sphingolipids bind TREM2 on myeloid cells and elicit antitumor responses in central nervous system cancers [35]. In present research, we found that TREM2 was primarily expressed in TAMs from GC patients and was negatively correlated with GC patients’ prognosis. Also, TREM2^+^ TAMs were identified as pro-tumor cells as they promote GC cell proliferation and migration. Besides, TREM2 leads to macrophages polarizing towards M2 phenotype and paucity of M1-like TAMs, therefore constructing an immunosuppressive TME, as M2-like TAMs expressing anti-inflammatory cytokines and chemokines, which decrease CD8^+^ T cell activation and enhance Treg recruitment, and contribute to tumor immune evasion [36].

Generally, TAMs promote cancer progression through secreting pro-tumor cytokines to promote tumor cell progression [37]. We confirmed that TREM2 overexpression promoted CCL8 secretion, which, in turn, contributed to tumor cell proliferation and migration. CCL8 exerts its effects by binding to receptors like CCR3, and CCR5 [38, 39]. It regulates cancer cell proliferation and migration by activating mTOR and ERK signaling pathways after binding to corresponding receptors [40, 41]. Overall, we discovered that TREM2 overexpression on TAMs promotes GC advancement, indicating TREM2’s role as a potent driver for GC progression via its interaction with cancer cells. In vivo and in vitro experiment proved that targeting CCL8 could efficiently disrupt TREM2^+^ TAMs mediated GC progression. Besides, we found that TREM2 overexpression contributed to an up-regulation of PD-L1 on TAMs. PD-L1 (CD274) is a type I transmembrane protein from the immunoglobulin (Ig) superfamily [42]. It interacts with PD-1 (CD279) on CD8^+^ T cells to suppress T cell proliferation, survival, cytokine generation, and other effector activities [43, 44]. TAM is the principal source of PD-L1 and exhibits supreme predictive value for immunotherapy in certain cancers [45, 46]. Our results suggest that TREM2^+^ TAMs might inhibit immune response through PD-L1. PD-L1^+^ TAMs inhibit immune response and induce immunotherapy resistance by impairing CD8^+^ T cell function and driving them into the exhaustion stage [47, 48]. Our data showed that the downregulation of PD-L1 on TAMs mediated by TREM2 knockdown resulted in less infiltration of exhausted CD8^+^ T cells in vivo. Moreover, to some extent, tumor-infiltrating immune cells, like Treg, myeloid-derived suppressor cells (MDSC), and TAMs, account for immune checkpoint inhibitor resistance. Shifting M2 like TAMs to M1 like TAMs can effectively increase the response to ICB therapy. Targeting PD-L1 on tumor cells or PD-L1^+^ exosome in serum can partially alleviate anti-PD-L1 therapy resistance [49–51]. Consistently, our results proved that, in an immune-competent mice model, knocking down TREM2 on TAMs potentiates the anti-PD-L1 therapy, as TREM2 knockdown on macrophages and anti-PD-L1 combined therapy significantly inhibits tumor growth and the accumulation of exhausted CD8^+^ T cells than anti-PD-L1 alone. Briefly, our work provides a new strategy to overcome anti-PD-L1 resistance for GC patients.

How TREM2 induces upregulation of CCL8 and PD-L1 draws our attention. We found that STAT1 directly binds to TREM2 in TAMs by mass spectrometry. STAT1 is a transcription factor belonging to the STAT protein family. It shifts into the nucleus to promote gene transcription after phosphorylated by kinase in cytoplasm [52]. It has been reported that STAT1 upregulates PD-L1 expression directly or by activating transcription factor IRF1 which is consistent with our results [53–55]. Besides, STAT1 is capable of regulating cytokines expression like CXCL9 [56]. Whether STAT1 activates CCL8 transcription is worthy of validating. Our results proved that p-STAT1 stimulated CCL8 transcription by binding to its promoter region 1(1909-1919) and 2(1743-1753). As shown in the results, TREM2 is positively related to the protein level of STAT1, but not at mRNA level. TREM2 preserved STAT1 stability even in the presence of the protein synthesis inhibitor cycloheximide. Moreover, the proteasome inhibitor MG132—but not the lysosomal inhibitor chloroquine—rescued the reduction of STAT1 caused by TREM2 knockdown, indicating that TREM2 maintains STAT1 stability through a proteasome-dependent mechanism. We found that TREM2 could reduce the TRIM21, a previously reported STAT1 E3 ligase [25], mediated proteasomal degradation of STAT1, thus elevating its protein level in TAMs. Additionally, we demonstrated that TREM2 stabilizes STAT1 by binding to its SH2 domain, thereby reducing the interaction between STAT1 and TRIM21. The intracellular domain of TREM2 is composed of a short cytosolic tail that lacks kinase function. TREM2 conducts signal mainly through SYK or PI3K by binding to DNAX activation protein (DAP) 12 or DAP10, respectively [57]. According to Serve et al., SYK stimulates acute myeloid leukemia cell proliferation by phosphorylating STAT3 and STAT5 at tyrosine 705 and 694 [58]. Therefore, we deduce that TREM2 phosphorylates STAT1 at tyrosine 701 through SYK rather than itself. Our results proved that, in this scenario, TREM2 binds to STAT1 to prevent its ubiquitination by TRIM21 and provides SYK a platform to phosphorylate STAT1; thereafter, phosphorylated STAT1 upregulates CCL8 and PD-L1 expression after its nucleus translocation.

The positive correlation between TREM2 and p-STAT1, CCL8, and PD-L1 was validated in clinical GC cohorts. More importantly, in GC patients, TREM2^+^ TAMs exclude CD8^+^ T cell infiltration and promote immune checkpoint expression on tumor-infiltrated CD8^+^ T cells, like PD-1, CTLA4, and TIM3, which shapes an inhibitory immune microenvironment. Thus, targeting TREM2^+^ TAMs might reshape the tumor immune microenvironment and enhance the immunotherapy efficacy.

## Conclusion

Our findings highlight the pivotal role of TREM2^+^ TAMs in driving GC progression and shaping an immunosuppressive tumor microenvironment. The TREM2/p-STAT1/CCL8 and PD-L1 axis in TAMs plays a crucial role in tumor progression and resistance to anti-PD-L1 therapy. Targeting TREM2 on macrophages represents a promising strategy to overcome anti-PD-L1 therapy resistance.

## Materials and Methods

### Clinical GC specimens

Cohort 1, composed of a total of 56 fresh GC specimens obtained from patients who were diagnosed with gastric adenocarcinoma at Zhongshan Hospital (Shanghai, China) from 2023 to 2024, was used to perform western blot (WB), real-time polymerase chain reaction (RT-qPCR) and immunohistochemistry (IHC). Zhongshan Cohort, composed of 135 pairs of GC and para-tumor specimens obtained from GC patients at Zhongshan Hospital from 2010 to 2012, was used to perform multiplex Immunofluorescence (mIF). The Zhongshan Flow Cytometry (ZSFC) cohort, composed of 60 GC specimens obtained from GC patients at Zhongshan Hospital from 2018 to 2019, was used to estimate immune checkpoint markers by corresponding flow cytometric data from each patient. The Zhongshan Hospital Research Ethics Committee approved this study, written informed consent was obtained from all patients, and the study was conducted in accordance with the Declaration of Helsinki.

### Animal studies

C57BL/6 mice at 5-6 weeks of age supplied by GemPharmatech (Nanjing, Jiangsu, China) were used for tumor xenograft experiment and assigned randomly into different treatment groups. Mice received a subcutaneous injection in the right flank with 100 μl of PBS (Cat. No. G4202, Servicebio Technology Co., Ltd., Wuhan, China) containing 1 × 10^7^ YTN-16 cells. Tumor volume was calculated according to the standard formula: *V* = 0.5×length × (width)^2^, where length represents the longest dimension and width the shortest dimension of the tumor. To eliminate the influence of host macrophages during tumor development, mice were treated with intravenous injections of clodronate liposomes 24 hours before subcutaneous tumor cell inoculation and every 4–5 days thereafter. Bone marrow cells were flushed from femurs and tibias of mice and cultured in the presence of M-CSF for 7 days to generate bone marrow-derived macrophages (BMDMs). Bone marrow-derived macrophages (BMDMs) were polarized into TAM-like macrophages by treatment with IL-4 (20 ng/mL) and IL-13 (20 ng/mL) for 48 hours, followed by transfection with TREM2 siRNA. TAM-like macrophages transfected with siTREM2 or control siRNA were delivered into subcutaneous tumor-bearing mice at a dose of 1×10⁶ cells in 100 μL PBS. For the in vivo CCL8 recuse experiment, C57BL/6 mice with GC tumors were injected with 200 μg of mouse recombinant CCL8 (Cat. No. HY-P7239; MCE) or isotype control twice a week for two weeks. For combination treatment, C57BL/6 J mice with GC tumors were randomly assigned into four groups (six animals each), given an-PD-L1 antibody (Cat. No. BE0101; Bio X Cell) and/or siTREM2 BMDMs. Mice were given intraperitoneally with 200 μg anti-PD-L1 antibody or isotype control twice a week for two weeks. At the end of the experiments, mice were sacrificed, tumors were excised for further analysis. All mice were kept in specified pathogen-free settings and routinely monitored. All anesthesia and euthanasia methods followed the Animal Research: Reporting of In Vivo Experiments (ARRIVE) criteria, and the study was approved by the Institutional Animal Care and Ethics Committee of Zhongshan Hospital, Fudan University.

### Public database analysis

To identify the TREM2 expression, a single-cell RNA sequencing dataset GSE183904 was included in this study. Data dimensionality reduction was achieved using principal component analysis (PCA). The nearest neighbors were located using the harmonized dimensions, and cell clusters were found at a resolution of 0.8. The dimensionality of was reduced by UMAP. ‘FindAllMarkers’ in the R package ‘Seurat’ was applied to distinguish specific markers for each cell cluster. According to specific markers, the cell clusters were identified through online database (http://bio-bigdata.hrbmu.edu.cn/CellMarker/). TREM2 expression and location were visualized by ‘FeaturePlot’ in the R package ‘Seurat’. The Stomach adenocarcinoma cohort (STAD) in The Cancer Genome Atlas (TCGA) database was used to explore the TREM2 mRNA level between the tumor and the para-tumor in GC. The correlation between TREM2 and GC patients’ prognosis was estimated in the TCGA and GSE15459 database. In addition, correlations between gene expression levels were assessed by Spearman correlation analysis.

### Cell culture and macrophage polarization

THP-1 and HEK293T were obtained from the Liver Cancer Institute, Zhongshan Hospital, Fudan University. AGS-1, MKN-45, MKN-47, HGC-27, and NCI-N87 were obtained from the Gastric Cancer Center, Zhongshan Hospital, Fudan University. Mouse GC cell line YTN-16 was granted from Prof. Sachiyo Nomura, University of Tokyo. YTN-16 and HEK-293T cells were cultured in Dulbecco’s modified Eagle’s medium (DMEM, Cat. No. C11965500BT, Gibco, Thermo Fisher Scientific, MA, USA), the human GC cell lines and THP-1 cells were maintained in Roswell Park Memorial Institute (RPMI, Cat. No. C11875500BT, Gibco) 1640 medium. All media were supplemented with 10% fetal bovine serum (Cat. No. 10099141 C, Gibco) and antibiotics (100 U/L penicillin and streptomycin, Cat. No. C0223, Beyotime). The culture was maintained in a 5% CO_2_ atmosphere at 37 °C with saturated humidity. THP-1 was polarized towards M0 macrophages by adding 100 ng/ml PMA (A606759, Sangon Biotech, Shanghai, China) for 24 hours of stimulation. The cells were then stimulated into M2 macrophages for 48 hours with 25 ng/ml IL-4 (11846-HNAE, Sino Biological) and 25 ng/ml IL-13 (10369-HNAE, Sino Biological).

### Co-culture assays

A sterile insert with a 0.4 µm pore polycarbonate membrane (Cat. No. 3412; Corning Inc., MA, USA) was employed for the in vitro co-culture system with GC cells and THP-1 induced tumor-associated macrophages. M2 macrophages (1 × 10^5^ cells/well) were polarized in the lower chamber, whereas GC cells were put in the top chamber. TAMs were obtained after M2 macrophages cocultured with GC cells for 24 hours. The cells and conditional media were subsequently preserved for future studies.

### Colony formation assay

300 GC cells were seeded per well in six-well plates and incubated for 14 days. After incubation, cells were fixed in PFA (Cat. No. P0099, Beyotime) for 30 minutes and stained with 1% crystal violet (Cat. No. C0121, Beyotime) for an hour. Prior to photography, each plate was cleansed adequately with pure water three times. The number of cells was counted by Image J software according to the protocol.

### 5-Ethynyl-2′-deoxyuridine (EdU) assay

To perform the 5-Ethynyl-20-deoxyuridine test (EdU), 1 × 10^4^ cells were planted in each well of 24-well plates. After 24 hours of culture, cells were incubated with 5-ethynyl-20-deoxyuridine (EdU; RiboBio Co., Ltd., Guangzhou, China) for 4 hours. After incubating with 1x Apollo reaction cocktail for 30 minutes, the cells’ DNA was stained with Hoechst 33342 (5 mg/mL) in each well for 20 minutes. Cells were imaged using a fluorescent microscope. To test the degree of correlation of the mitotic index, the S-phase fraction was utilized as a measure of cell proliferation, as previously stated [59].

### Transwell assay

The upper chambers of a transwell plate (Cat. No. 3412; Corning) were coated with or without matrigel for the invasion and migration assay, respectively. For migration and invasion assays, roughly 10^5^ or 2 × 10^5^ cells were plated in upper chambers and cultivated for 24 hours. The lower compartment was filled with 700 µL of RPMI-1640 with 10% FBS, whereas the top chamber was filled with RPMI-1640 without FBS. Every chamber was rinsed with PBS three times. The chambers were submerged in PFA for 30 minutes to fix cells. Chambers were then soaked in 1% crystal violet for an hour before being washed thrice with PBS.

### Wound healing assay

5 × 10^5^ cells were seeded onto 6-well plates, and a cell monolayer was formed after 8 hours of incubation. The monolayer was lined out with pipette tips, leaving an equal gap in the center. The cells were rinsed in PBS and grown in RPMI-1640 with 1% FBS for 24-48 hours at 37°C in an incubator. Images were captured every 12 hours. Image J was utilized to determine the wound distance.

### RNA extraction and quantitative real-time PCR

The FastPure® Cell/Tissue Total RNA Isolation Kit V2 (Cat. No. RC112-01, Vazyme Biotech Co., Ltd., Nanjing, China) was used to separate total RNA from cells according to the procedure. RNA was reverse-transcribed into cDNA using HiScript® III All-in-One RT SuperMix Perfect for qPCR (Cat. No. R333-01, Vazyme). The QuantSudio™ 3 real-time PCR equipment (Applied Biosystems, Thermo Fisher Scientific) and ChamQ Universal SYBR qPCR Master Mix (Cat. No. Q311-02, Vazyme) were used to perform quantitative analysis, following manufacturer instructions. The expression of GAPDH was used as an internal reference. The primers sequences used for the qPCR were provided in Table S1.

### Flow cytometry

The cell suspensions were first incubated with BD Fc block (BioLegend, CA, USA) for 30 minutes on ice, followed by incubation with relevant antibodies in the FACS buffer (5% FBS in PBS), including APC-CD3, FITC-CD8, PE-CD8, PD-1-BV421, FITC-F4/80, CD11b-PerCP Cy5.5, APC-CD86, APC-CD206 (BioLegend) for 30 minutes on ice or at 2–8°C. The cells were washed with 1 mL flow cytometry staining solution and centrifuged at room temperature 400–600 × *g* for 5 min. The supernatant was discarded, and the cells were re-suspended with 500 μl PBS, followed by detection and analysis. Cell subsets were analyzed: CD8^+^ T cells, gated as CD3^+^/CD8^+^ cells; Macrophage cells, gated as F4/80^+^/CD11b^+^ cells or CD11b^+^ cells and subdivided into CD86^+^(M1) and CD206^+^(M2). All data were collected using a BD FACS Aria II flow cytometer and analyzed with FlowJo software (Tree Star, California, USA). Details of gating were provided in Fig. S9

### Western blot

Western blotting was done as previously reported [60]. Related equipment and reagents were purchased from Epizyme Biotech, Shanghai, China. Detailed information on primary antibodies used in the study was provided in Table S2.

### Co-immunoprecipitation

The cells were washed twice with PBS. Cell lysates were prepared by adding lysis buffer in a ratio of 20 µL per 1 × 10^5^ cells, with the addition of protease inhibitor (1:100). The mixture was incubated on ice for 20 min and then centrifuged at 12,000 *g* for 10 min at 4°C. The supernatant was collected. Co-IP was performed following the protocol provided by Classic Protein A/G Immuno-Precipitation Kit (YJ201, Epizyme Biotech, Shanghai, China). Antibodies and IgG were added to 250 µL lysis buffer, respectively, to form a protein-antibody complex after incubated at 4 °C on a rotating mixer overnight. Protein A/G magnetic beads were thoroughly suspended, and 25 µL of beads were resuspended in 250 µL of lysis buffer twice, and magnetic beads were added to the protein-antibody complex, and the mixture was incubated at 4 °C on a rotating mixer for 6 hours. The beads-antibody-protein complex were washed four times with 200 µL washing buffer and the supernatant was discarded. 50 µL of 1 × SDS-PAGE loading buffer was added, mixed thoroughly, and heated at 95 °C for 10 min, and the beads were separated. The supernatant was collected for subsequent SDS-PAGE analysis.

### Glutathione S-Transferase (GST) precipitation assays

GST, GST-TREM2, and GST-STAT1 plasmids were transfected into the Escherichia coli BL21 strain (DE3). After overnight culture, GST fusion proteins were lysed from the bacteria. Pull-down binding assays were based on the instructions of Pierce™ GST Protein Interaction Pull-Down Kit (Cat. No. 21516, Thermo Fisher Scientific) [61].

### ELISA

Conditioned media from the cultured TAMs were collected. ELISA assays were performed using the Human MCP-2/CCL8 ELISA Kit (Cat. No. EHCCL8, Thermo Fisher Scientific) according to the manufacturer’s instructions [61].

### Immunofluorescence staining

TAMs were prepared as described above and subsequently rinsed with PBS three times. Samples were fixed and blocked by specific reagents (Cat. No. P0098, P0260, Beyotime). The samples were then incubated with the corresponding primary antibody overnight at 4 °C. The fluorophore-conjugated secondary antibody was added and incubated for 1 hour at room temperature. The nuclei were stained with Hoechst 33258 (Cat. No. C1011, Beyotime) for 15 mins. Images were collected and processed with the Olympus confocal microscope and ImageJ software.

### Luciferase reporter assay

The cells were transfected with Lipofectamine™ 3000 Transfection Reagent (Cat. No. L3000001, Invitrogen, Thermo Fisher Scientific) containing truncated or mutant CCL8 promoter luciferase reporter and thymidine kinase promoter-Renilla luciferase reporter plasmid for normalization. After 48 hours, luciferase activity was assessed using a dual-luciferase reporter system (Cat. No. 16186, Pierce™ Renilla-Firefly Luciferase Dual Assay Kit, Thermo Fisher Scientific), according to the manufacturer’s instructions [62].

### Chromatin immunoprecipitation (ChIP) assay

STAT1 knockdown TAMs were crosslinked with 1% formaldehyde in PBS for 10 minutes at room temperature. Glycine solution (1X) was added and incubated for 5 mins at room temperature. Chromatin DNA was digested by MNase and sonicated before being treated with anti-STAT1 or IgG antibody. Chromatin coupled to STAT1 was precipitated with ChIP Grade Protein A/G magnetic Beads. Beads were then eluted by elution buffer (1X) containing NaCl and Proteinase K. DNA was recovered after several rounds of centrifugations by DNA column elution solution. The precipitated DNA was examined using qPCR to identify potential STAT1 binding sites in the CCL8 promoter region. Products were supplied by Pierce™ Magnetic ChIP Kit (Cat. No. 26157; Thermo Fisher Scientific).

### Mass spectrometry

Anti-TREM2 antibody immunoprecipitants were produced by co-ip assay, put onto an SDS-PAGE gel, and separated by electrophoresis. The gels were then sliced into slices based on the protein markers and hydrolyzed to make peptides. Later, the peptides were identified using mass spectrometry on an Orbitrap Elite mass spectrometer and a scan of the fragment spectra against the UniProt database.

### RNA sequencing

Total RNA was isolated from TAMs in tumor or para-tumor tissue, as well as THP-1-induced TAMs and cDNA libraries were created. Sequencing was carried out using the BGISEQ 500 platform, and libraries were sequenced single-end. The sequenced segments were compared to the human reference genome using HISATt2 software, and the results were translated into binary alignment map (BAM) files using HISAT2. Using Stringtie’s default parameters, we estimated the exon model per kilobase base fragment per million gene location fragments (FPKM) for each discovered gene. DEGs were identified using DESeq2 with the criterion of multiple variants >1 and adjusted p-value < 0.05.

### Statistical analyses

The sample size for each experiment was determined according to our prior experience and reference to published literature. All collected data were included in the analyses, and no data were excluded. All quantitative data are presented as mean ± SD. Statistical analysis using GraphPad Prism revealed significant differences between groups (*: *p* < 0.05, **: *p* < 0.01, and ***: *p* < 0.001). Two groups comparison was compared by student’s t-test. Multiple group comparison was conducted by one-way analysis of variance (ANOVA). The fraction of IHC intensity between tumor and normal tissue is compared using the Chi-square (χ²) test. For survival analysis, the log-rank test in conjunction with the Kaplan-Meier methods was employed. Spearman’s correlation analysis was used to examine the relationships between the variables.

## Supplementary information

Supplemental Figures

Supplemental Tables

Supplemental Material

## Data Availability

The data supporting this study’s findings are available from the corresponding author upon reasonable request.
