# Supplementary material for: TREM2 facilitates gastric cancer progression and immune evasion via inhibiting TRIM21-mediated STAT1 degradation in tumor-associated macrophages
Source: Cell Death Dis. 2025 Nov 18;16(1):845. doi: 10.1038/s41419-025-08198-4 (PMC12627648; doi:10.1038/s41419-025-08198-4)
Supplement: Supplementary file 1 — Supplemental Figures [file 41419_2025_8198_MOESM1_ESM.docx]

**Supplemental Figures**


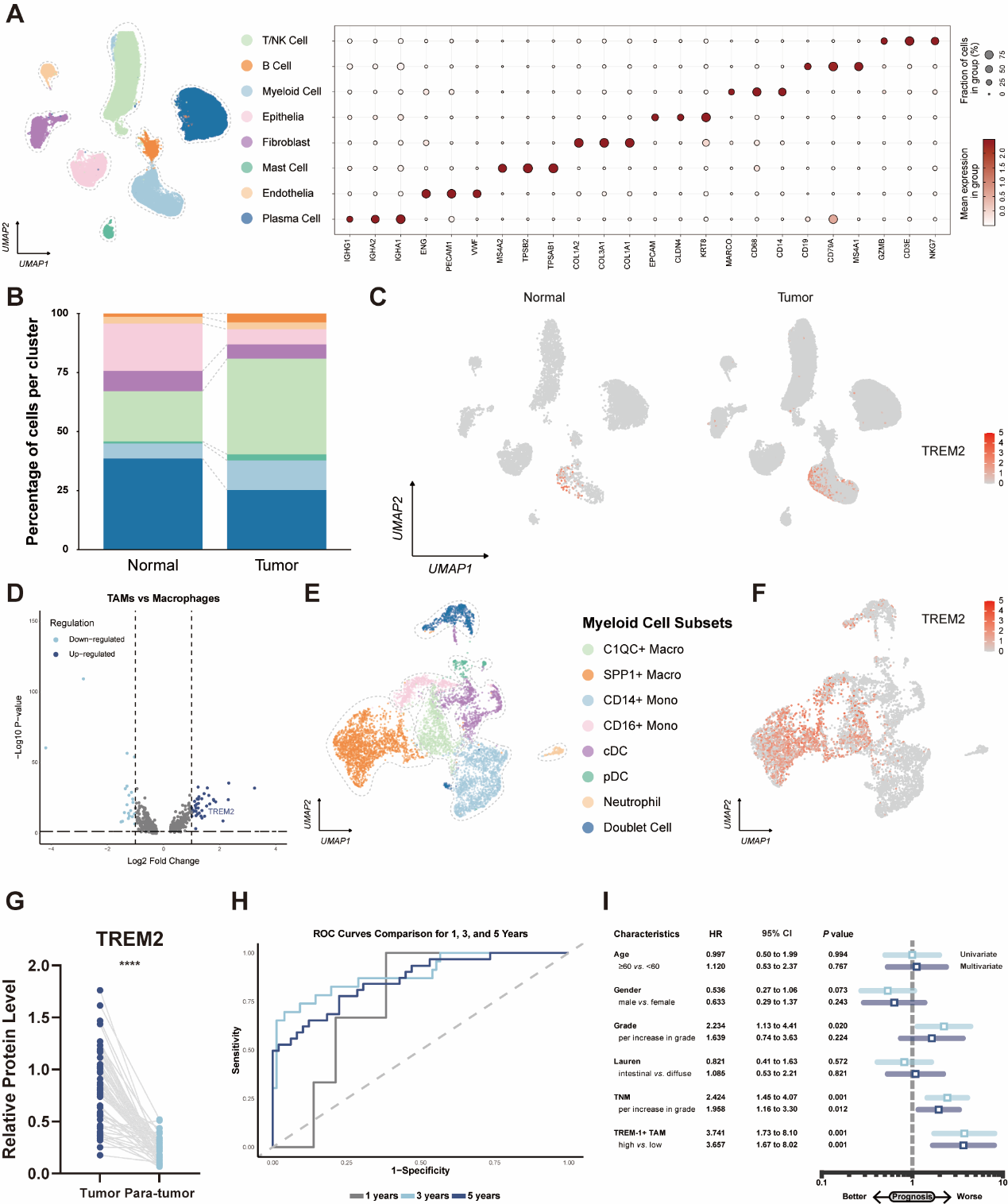


**Figure.S1 TREM2 is a prognosis predictor for GC patients**

(A) Uniform Manifold Approximation and Projection (UMAP) of GSE183904 representing 8 unique cell states color-coded by their corresponding cell lineage (left panel) and specific markers of each cluster (right panel). (B) Cell proportion of different cells in gastric cancer and normal samples. (C) TREM2 expression profile in normal and tumor samples in GSE183904. (D) Volcano plot of DEGs detected in GSE183904 from TAMs and normal macrophages. (E) UMAP of myeloid cells in GSE183904 datasets, representing 8 unique subset clusters. (F) TREM2 expression in different myeloid subset clusters. (G) Dot plot showing the TREM2 expression in 56 pairs of GC samples and adjacent normal tissue. (H) The AUC values of time-dependent ROC curves verified the prognostic performance of the TREM2 expression in the Zhongshan Cohort. (I) Forest plot showing the results of univariate and multivariate analysis of factors associated with prognosis.


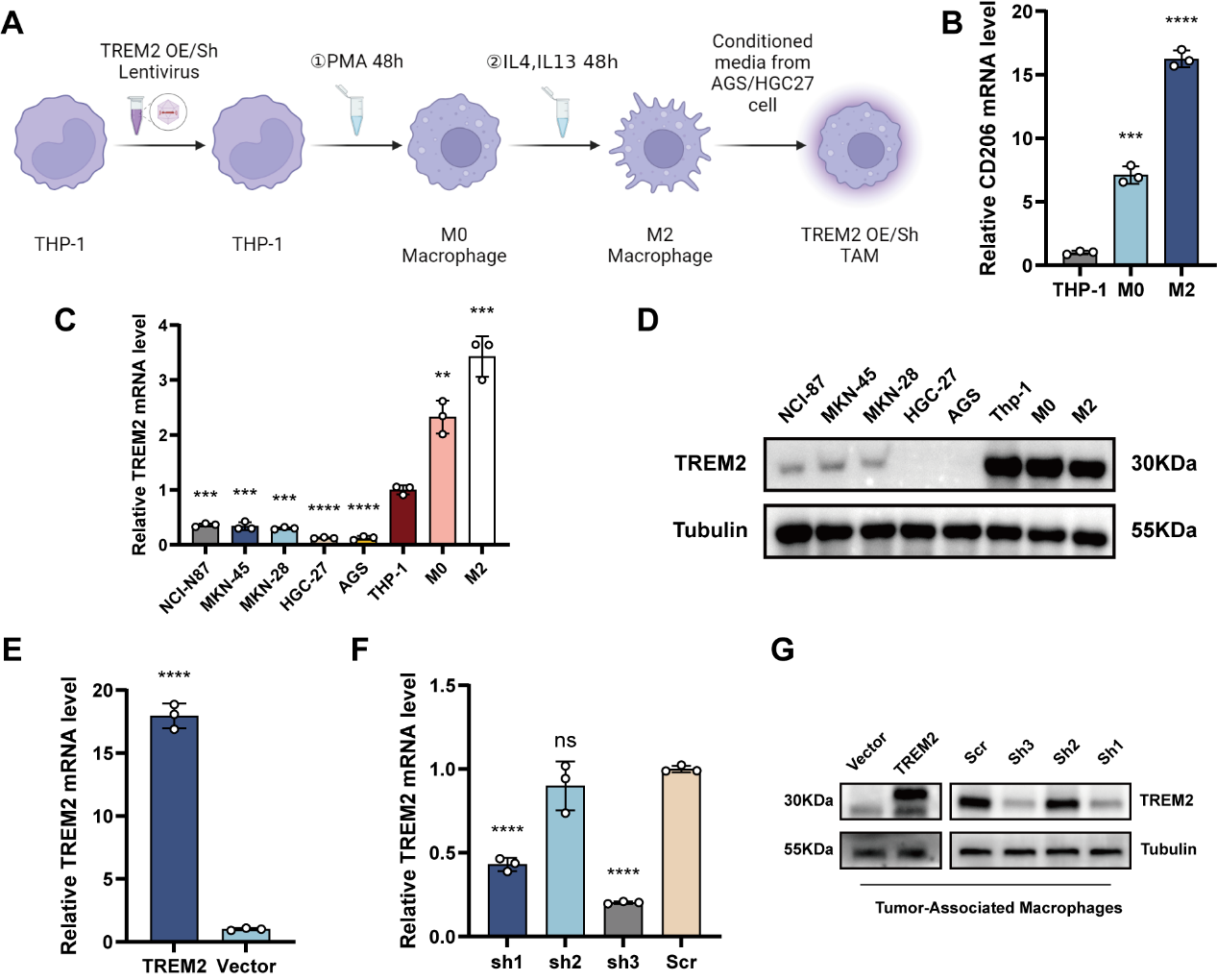


**Figure.S2 TREM2 promotes TAMs M2 polarization**

(A) Schematic diagram of constructing TREM2 overexpression and knockdown TAMs. (B) RT-qPCR analyzed the expression of the markers of M2-like macrophages. (C, D) RT-qPCR (C) and Western blot (D) showing the relative TREM2 expression in five GC cell lines, THP-1 and THP-1 induced macrophages. (E-G) RT-qPCR (E, F) and western blot (G) analyzed the expression of the TREM2 in indicated cells.


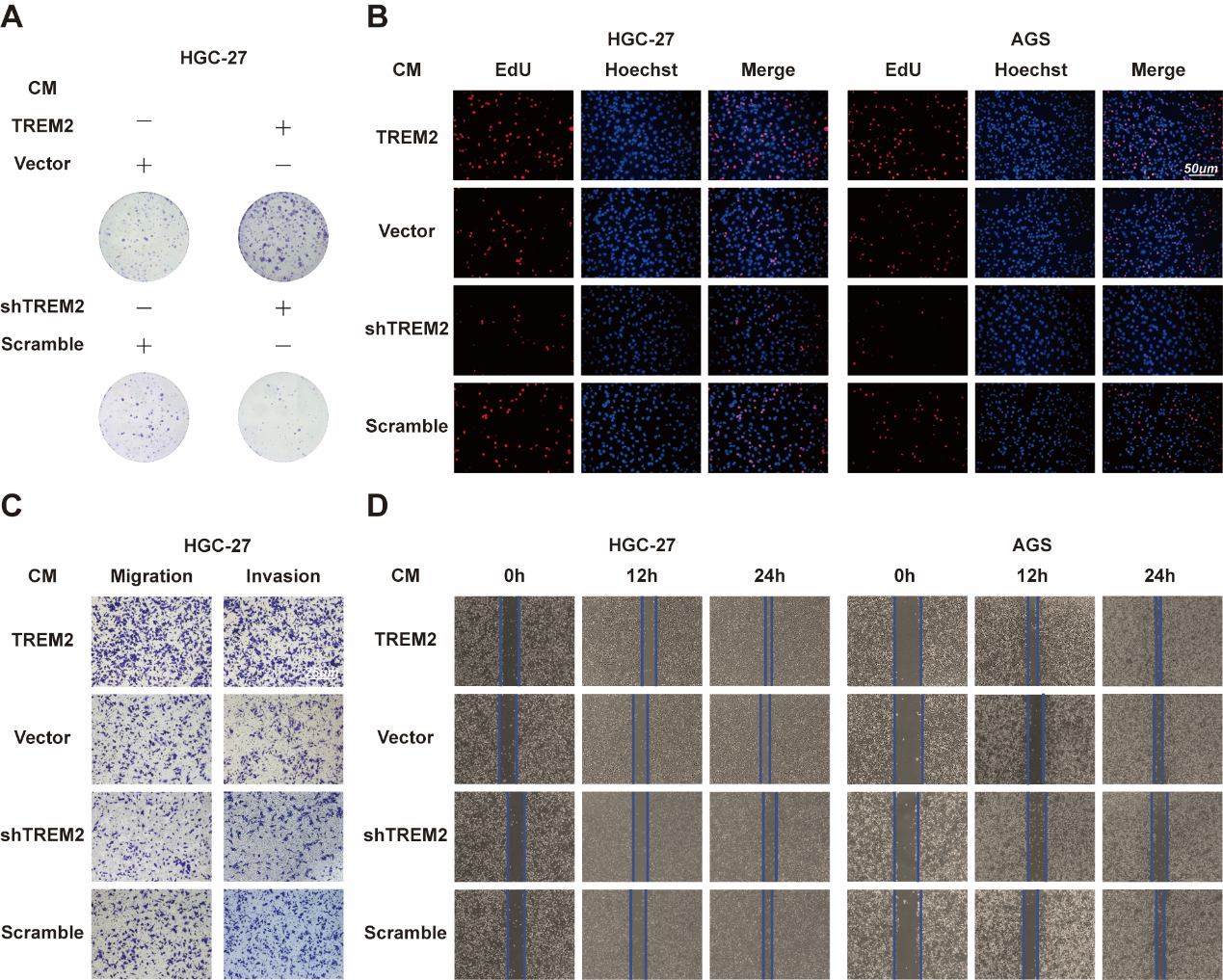


**Figure.S3 TREM2+ TAMs promote HGC-27 and AGS proliferation and migration**

The Colony formation assay (A) and the EdU assay (B) were used to detect the effect of CM from TAMs on HGC-27 and AGS proliferation. Transwell assay (C) and wound healing assay (D) were used to detect the effect of CM from TAMs on HGC-27 and AGS migration and invasion.


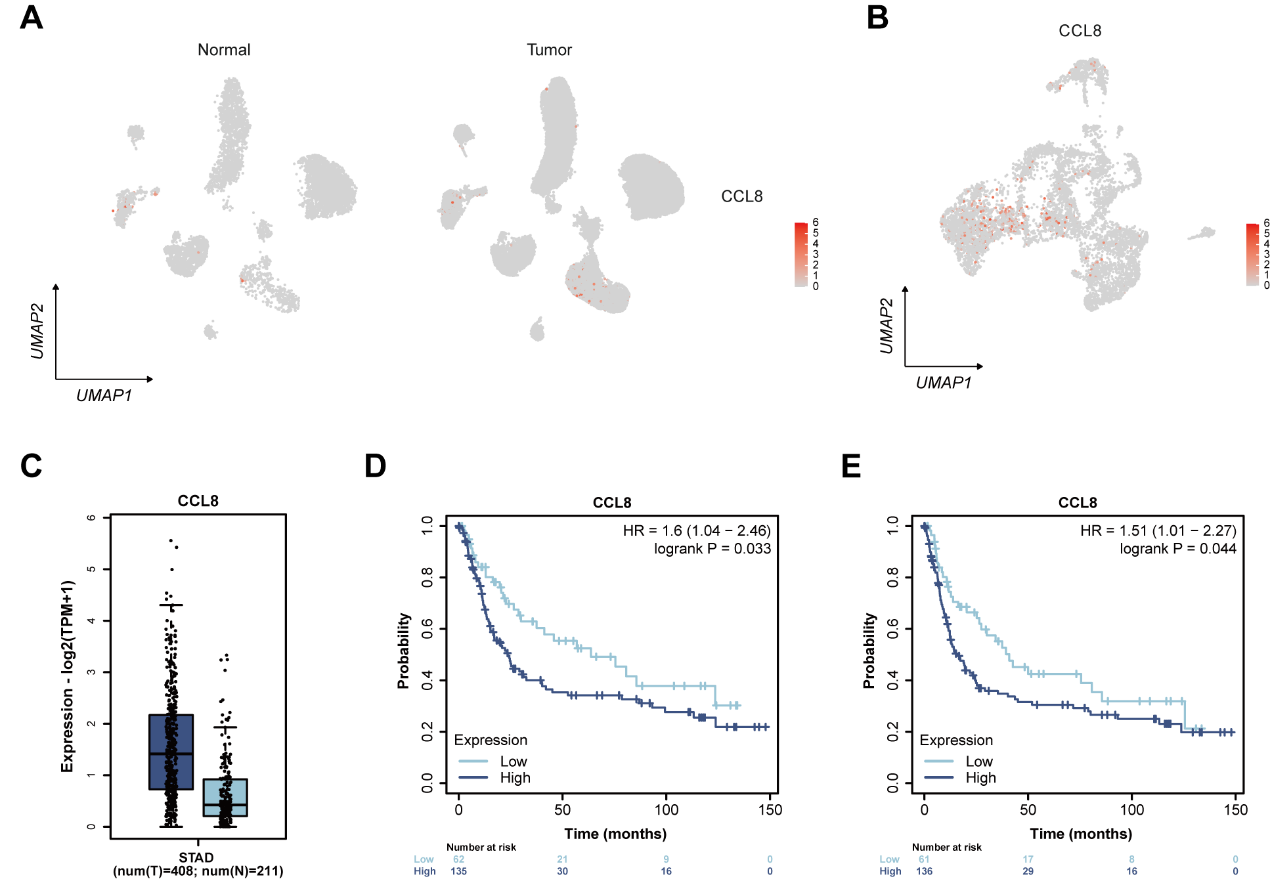


**Figure.S4 CCL8 expression profile in public datasets**

(A) GSE183904 GC single cell dataset analysis of CCL8 expression between tumor and normal sample. (B) CCL8 expression profile in myeloid cells. (C) TREM2 expression between GC tissue and normal tissue in TCGA datasets. (D-E) OS (D) and FP (E) curves based on TREM2 expression in patients with GC from GSE15459.


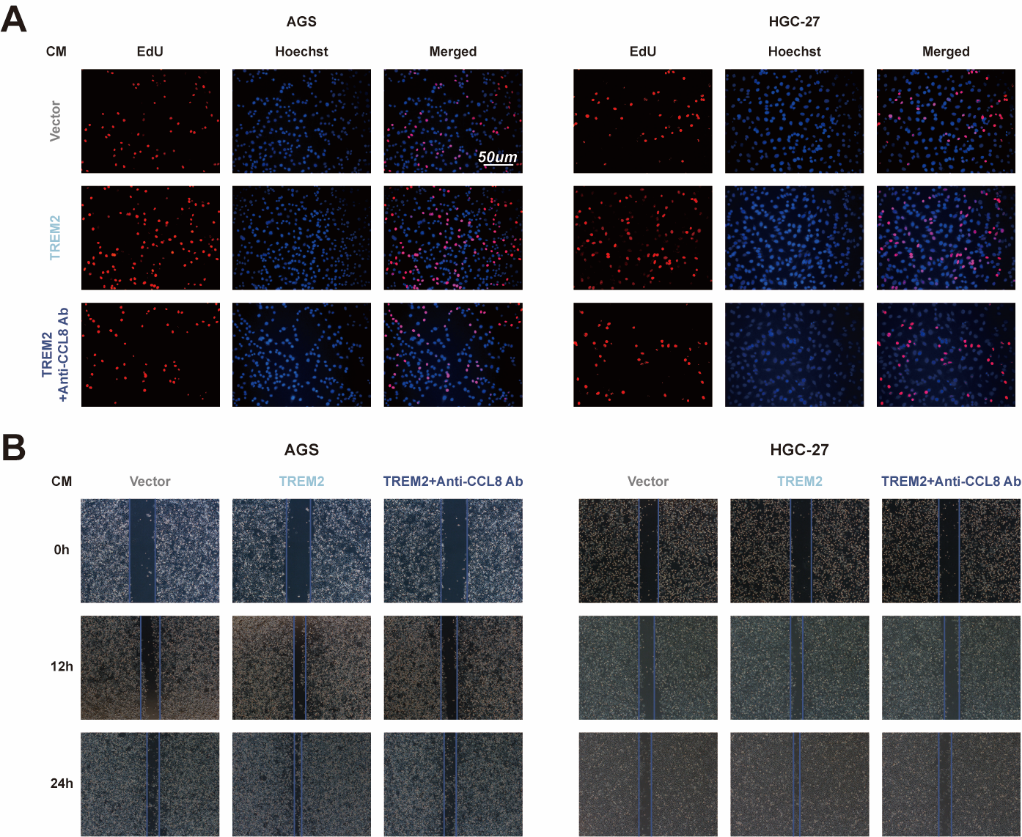


**Figure.S5 CCL8 antibody reverses the promotion effect for GC proliferation and migration mediated by TREM2-knockdown TAMs in vitro**

(A) EdU analysis for cell proliferation of GC cells cultured by CM from TREM2 TAMs, with or without anti-CCL8 Ab. (B) Wound-healing assays were used to test cellular migration of GC cells cultured by CM from TREM2 TAMs, with or without anti-CCL8 Ab.


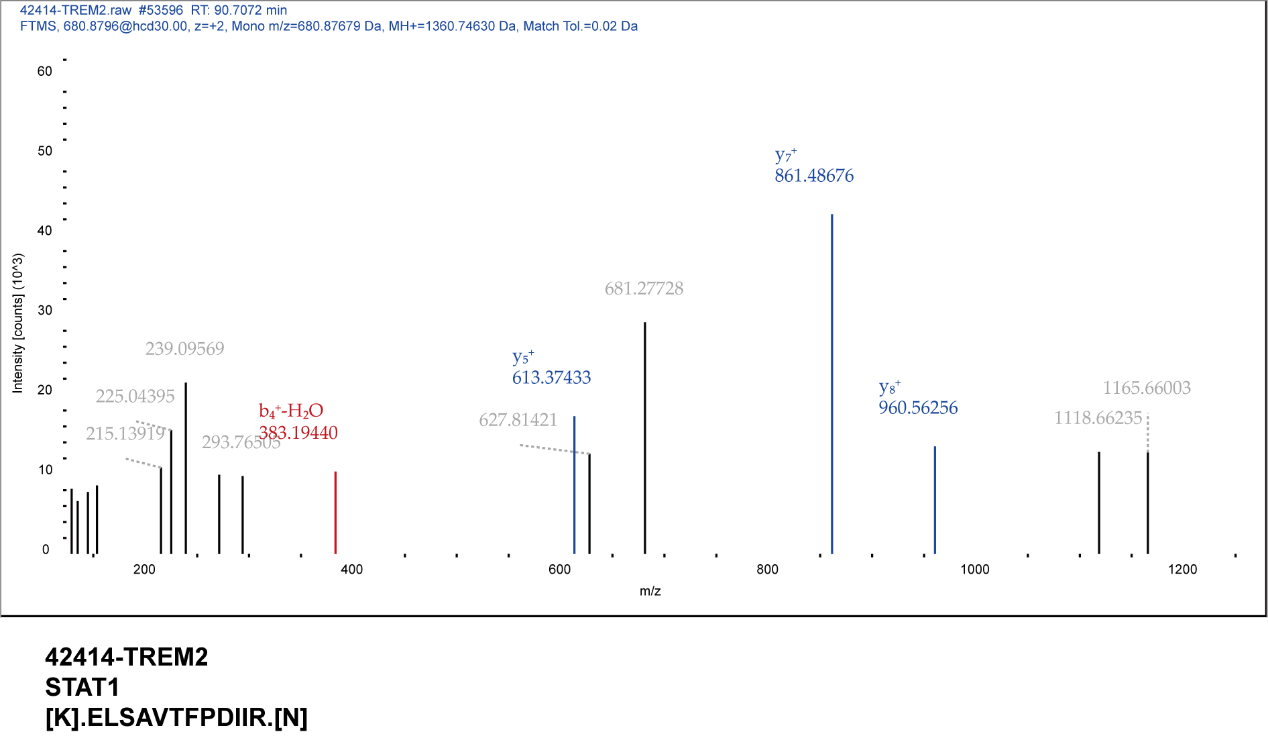


**Figure.S6** **Proteomic analysis of TREM2 complexes by LC-MS/MS**


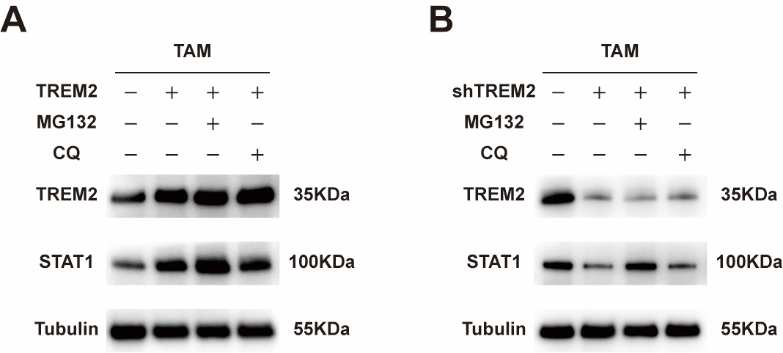


**Figure.S7 Western blot of TREM2, STAT1 and Tubulin in TREM2 overexpression (A) and knockdown (B) TAMs with MG132 or CQ treatment.**


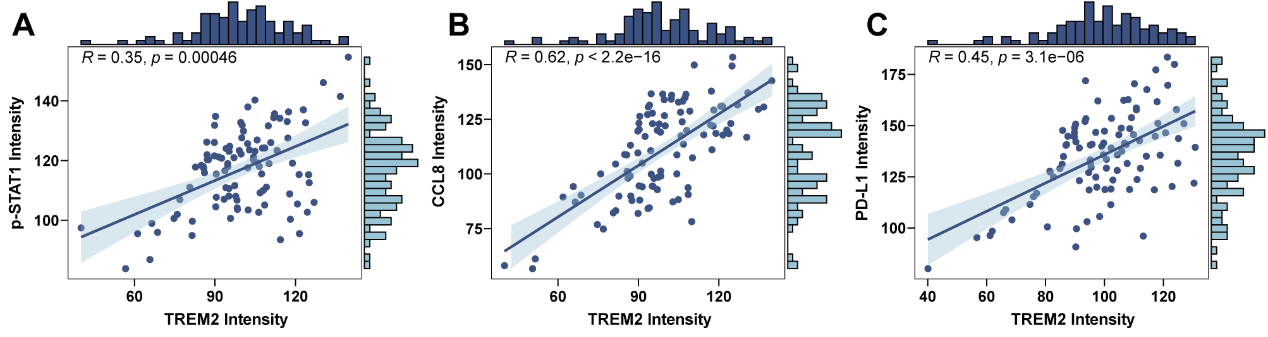


**Figure.S8 TREM2 positively related to p-STAT1, CCL8 and PD-L1 in TAMs**

(A-C) Correlation analysis between p-STAT1 (A), CCL8 (B), PD-L1 (C), and macrophage TREM2 expression in GC tissues.


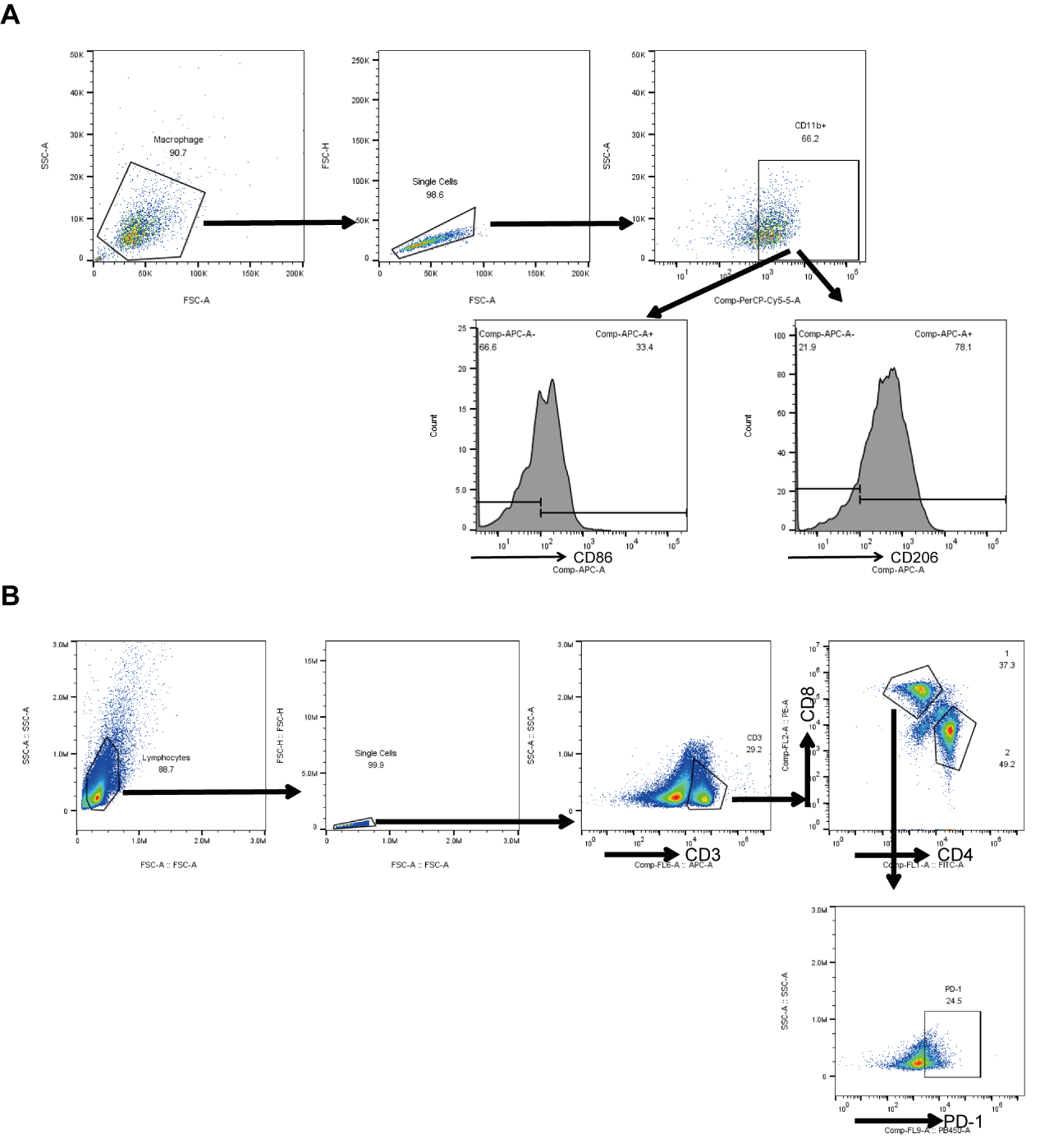


**Figure.S9 Flow cytometry gating strategy**

(A) CD86/CD206 flow cytometry gate strategy. (B) PD-1 flow cytometry gate strategy.
