# Supplementary material for: TREM2 facilitates gastric cancer progression and immune evasion via inhibiting TRIM21-mediated STAT1 degradation in tumor-associated macrophages
Source: Cell Death Dis. 2025 Nov 18;16(1):845. doi: 10.1038/s41419-025-08198-4 (PMC12627648; doi:10.1038/s41419-025-08198-4)
Supplement: Supplementary file 2 — Supplemental Tables [file 41419_2025_8198_MOESM2_ESM.docx]

**Supplemental Tables**

**Table S1 Summary of primer sequences**

| **Gene** | **Sequence** |
| --- | --- |
| GAPDH | Forward: CTGGGCTACACTGAGCACC |
|  | Reverse: AAGTGGTCGTTGAGGGCAATG |
| TREM2 | Forward: ACAGCATCTCCAGGGCTGA |
|  | Reverse: TGCCAGAGCAGAACAAGGAG |
| CCL8 | Forward: TGGAGAGCTACACAAGAATCACC |
|  | Reverse: TGGTCCAGATGCTTCATGGAA |
| PD-L1 | Forward: GGACAAGCAGTGACCATCAAG |
|  | Reverse: CCCAGAATTACCAAGTGAGTCCT |

**Table S2 Summary of primary antibodies for western blot**

| **Antibodies** | **Source** | **Identifier** |
| --- | --- | --- |
| Alpha Tubulin Mouse Monoclonal antibody | Proteintech | Cat. No. #66031-1-Ig |
| CCL8 Rabbit  Polyclonal Antibody | Signalway Antibody | Cat. No. #55062-1 |
| GAPDH (D16H11) XP®  Rabbit mAb | Cell Signaling Technology | Cat. No. #5174 |
| DYKDDDDK Tag (D6W5B) Rabbit mAb | Cell Signaling Technology | Cat. No. #14793 |
| HA-Tag (C29F4) Rabbit mAb | Cell Signaling Technology | Cat. No. #3724 |
| His-Tag (D3I1O) XP®  Rabbit mAb | Cell Signaling Technology | Cat. No. #12698 |
| Stat1 Antibody | Cell Signaling Technology | Cat. No. #9172 |
| Phospho-Stat1 (Tyr701) (58D6) Rabbit mAb | Cell Signaling Technology | Cat. No. #9167 |
| Syk (D3Z1E) XP®  Rabbit mAb | Cell Signaling Technology | Cat. No. #13198 |
| TREM2 (D8I4C)  Rabbit mAb | Cell Signaling Technology | Cat. No. #91068 |
| PD-L1 (E1L3N®) XP®  Rabbit mAb | Cell Signaling Technology | Cat. No. #13684 |
| Phospho-Tyrosine (P-Tyr-1000) MultiMab® Rabbit mAb mix | Cell Signaling Technology | Cat. No. #8954 |
