# Supplementary material for: TREM2 facilitates gastric cancer progression and immune evasion via inhibiting TRIM21-mediated STAT1 degradation in tumor-associated macrophages
Source: Cell Death Dis. 2025 Nov 18;16(1):845. doi: 10.1038/s41419-025-08198-4 (PMC12627648; doi:10.1038/s41419-025-08198-4)

Fig.1

TREM2

Tubulin

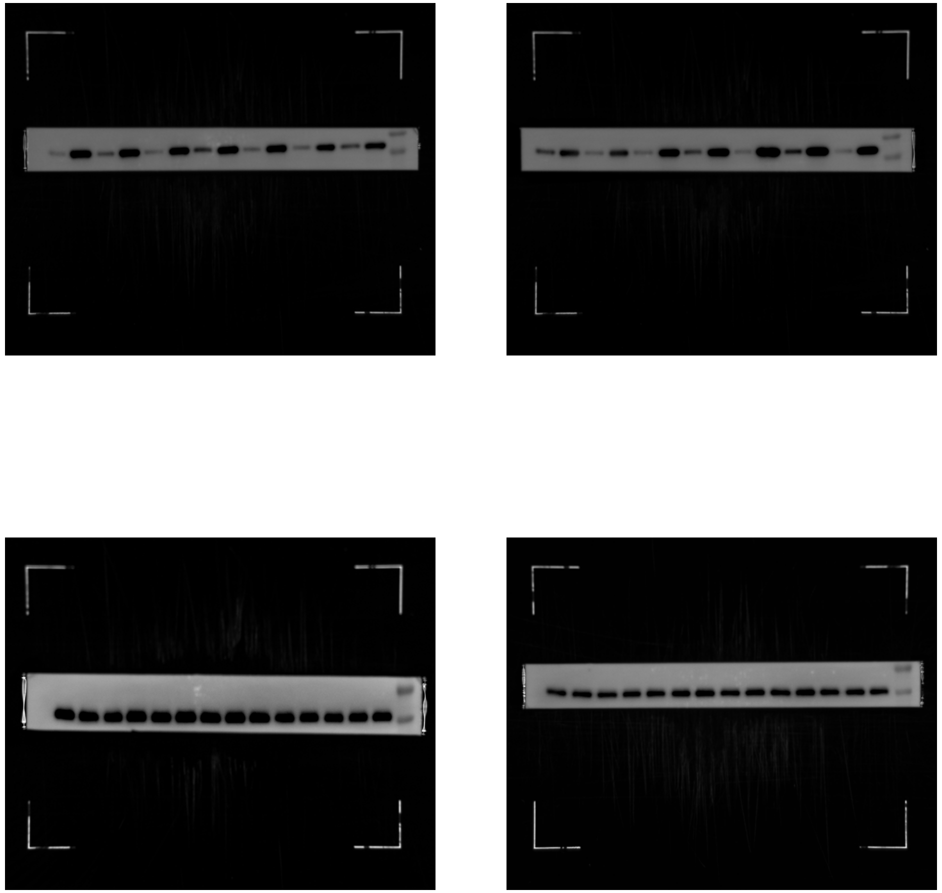

Fig.3

CCL8

GAPDH

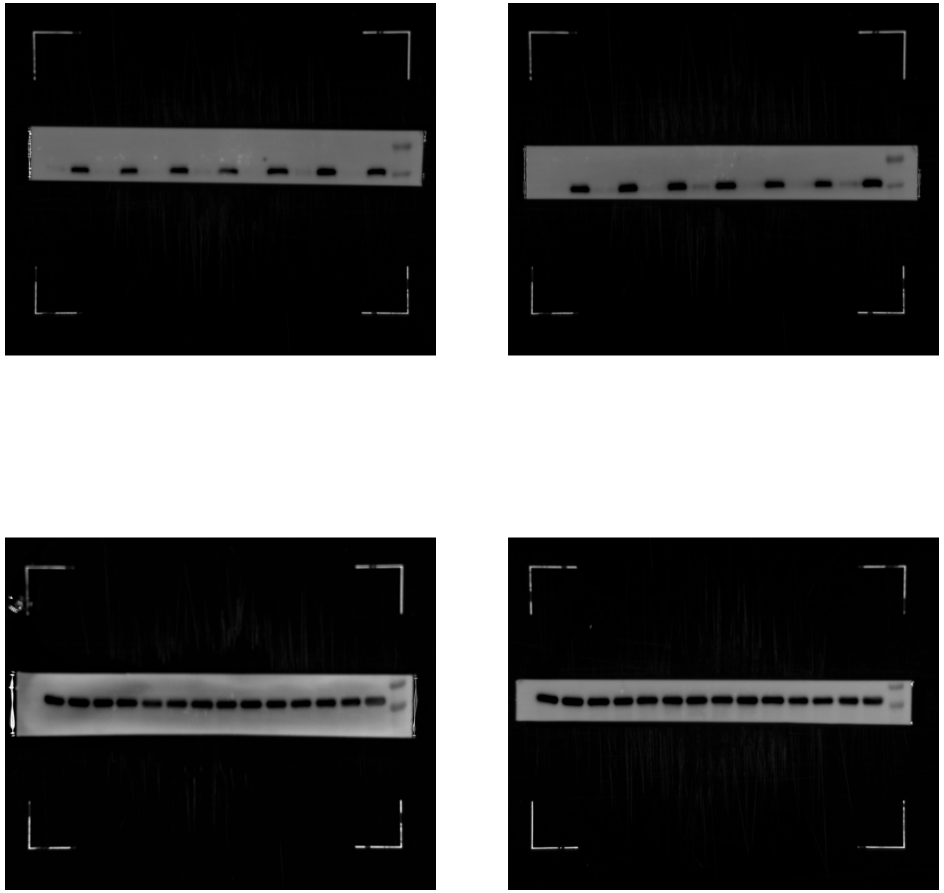

Fig.5D

STAT1

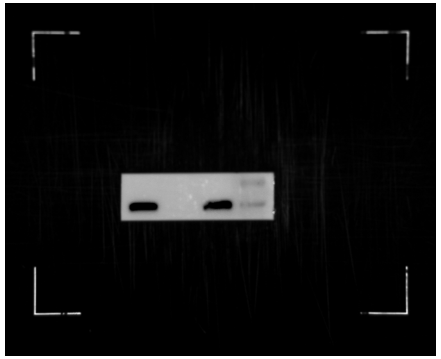

TREM2

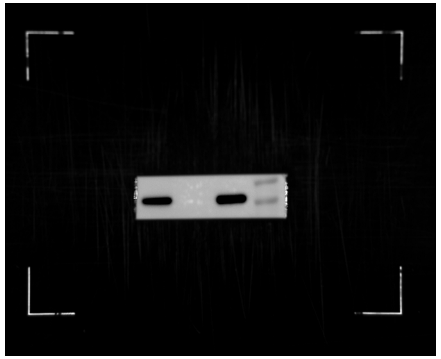

Fig.5E

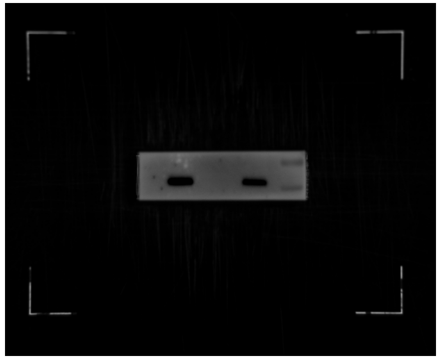

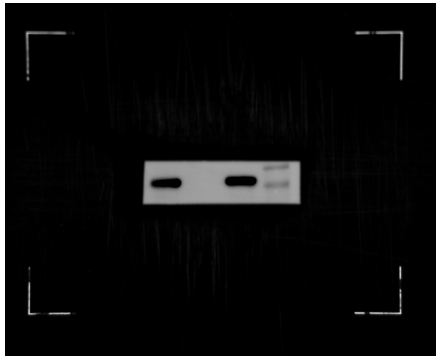

Fig.5G (Left)

Flag

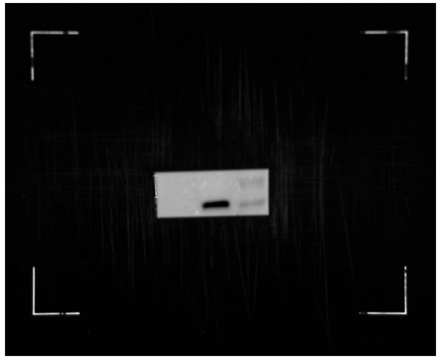

Flag

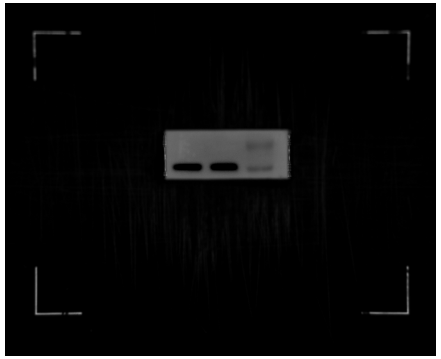

HA

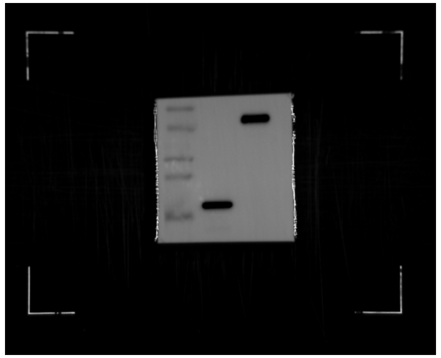

Fig.5G (Right)

Flag

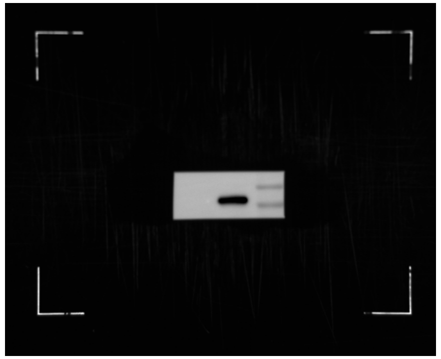

Flag

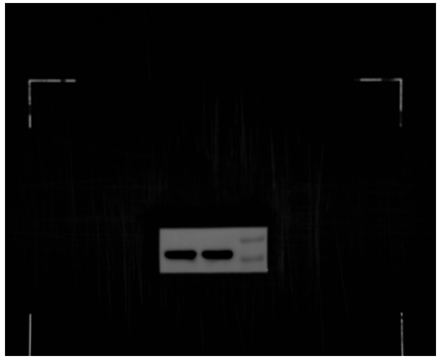

HA

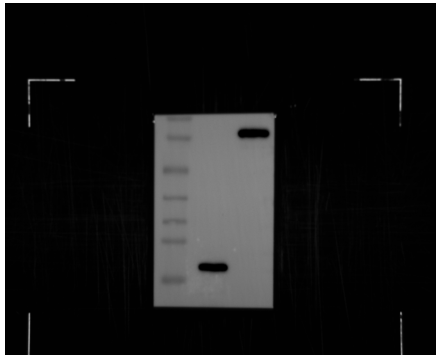

Fig.5H
(Left)

TREM2

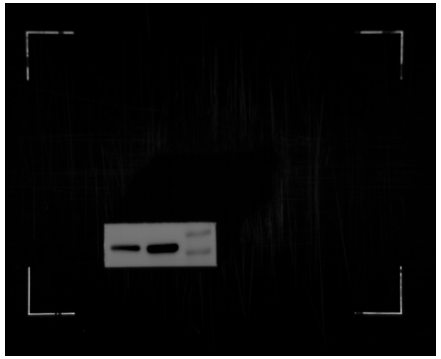

CCL8

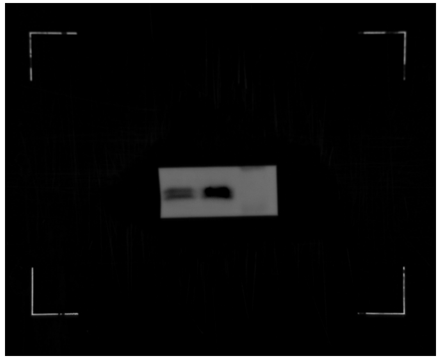

p-STAT1

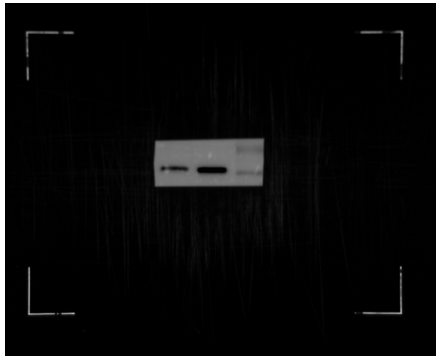

PD-L1

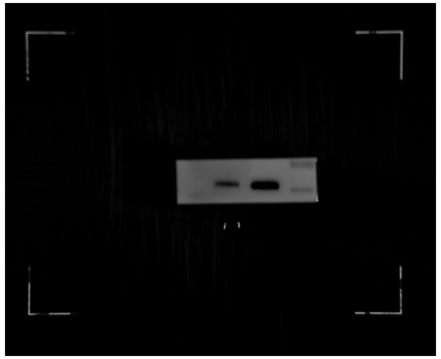

STAT1

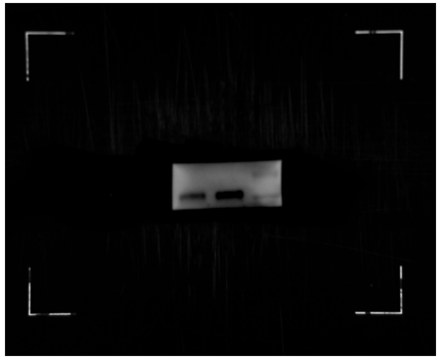

Tubulin

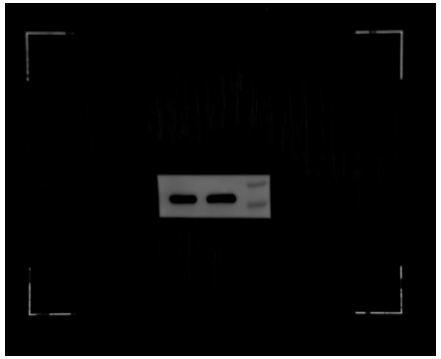

Fig.5H
(Right)

TREM2

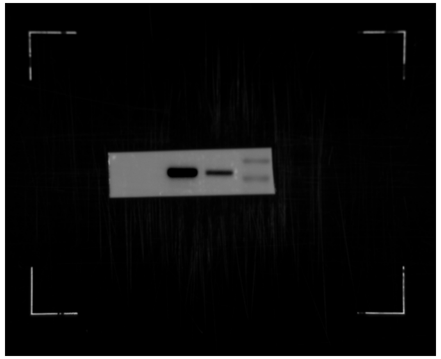

CCL8

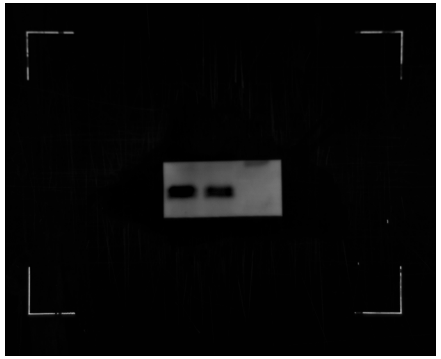

p-STAT1

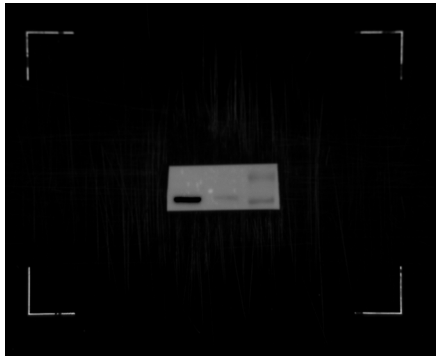

PD-L1

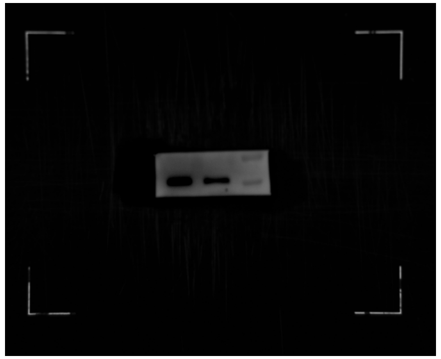

STAT1

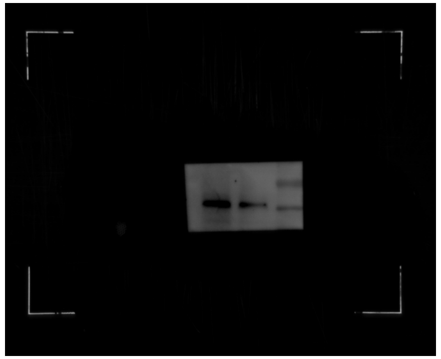

Tubulin

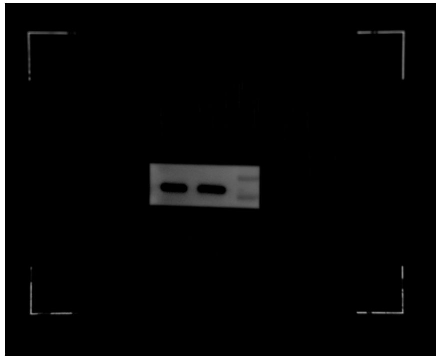

Fig.5I

CCL8

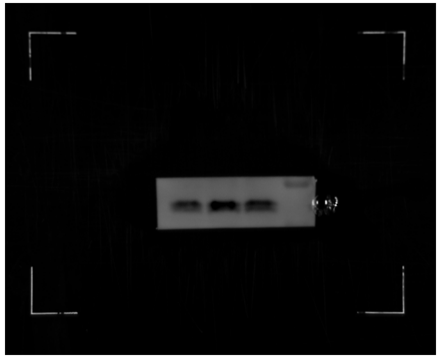

GAPDH

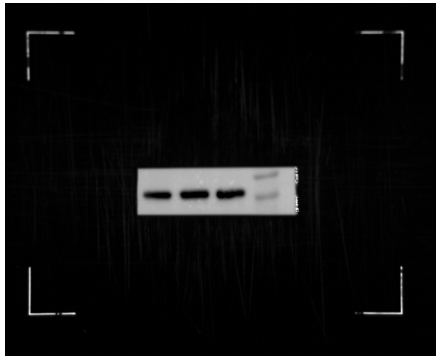

Fig.5J

CCL8

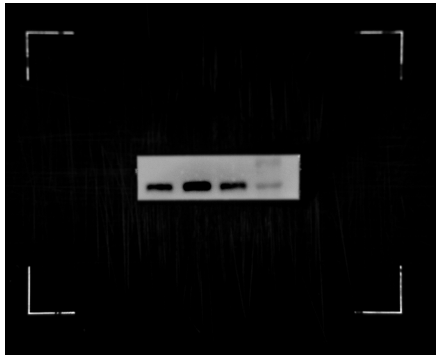

GAPDH

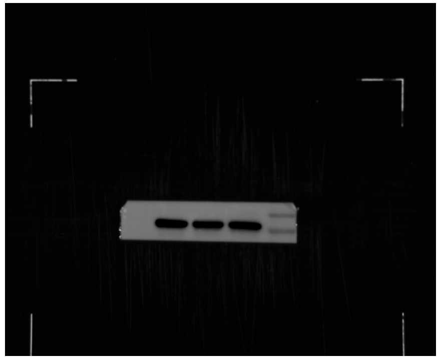

Fig.6B

shTREM2 Scr TREM2 Vec

STAT1

GAPDH

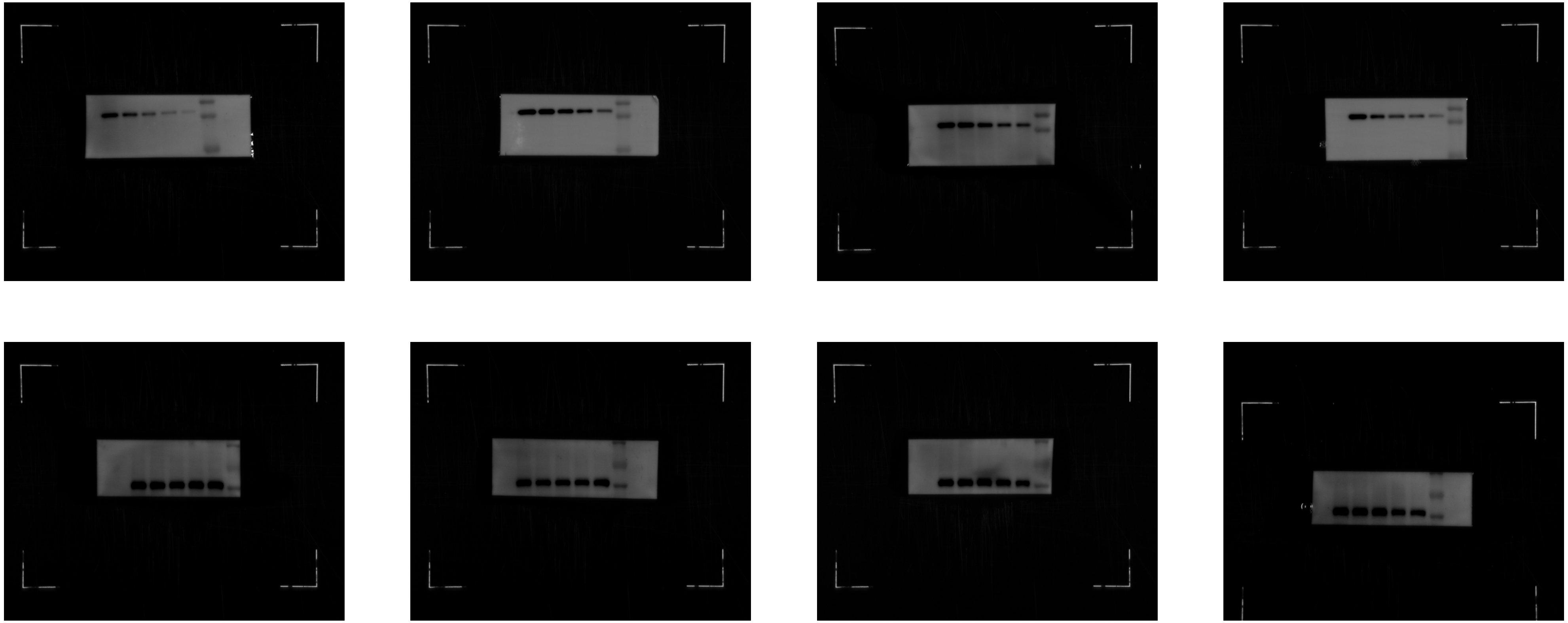

Fig.6C

TREM2

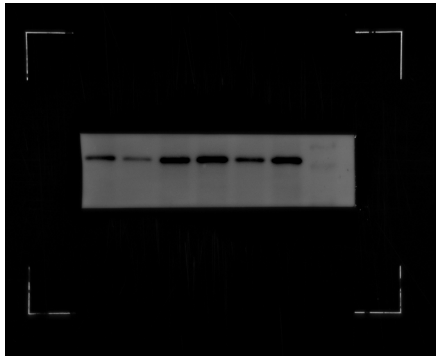

STAT1

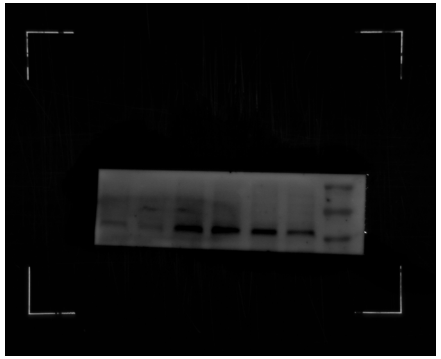

Tubulin

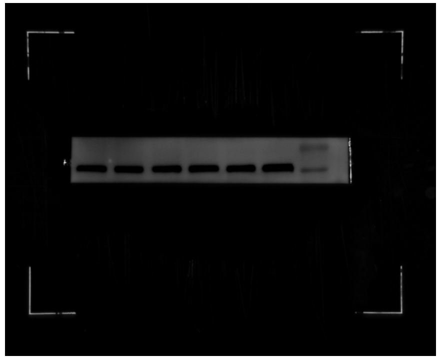

Fig.6D

Ub

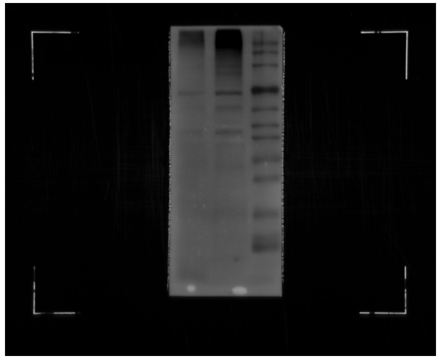

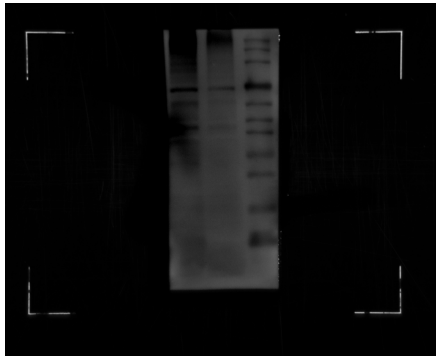

TREM2

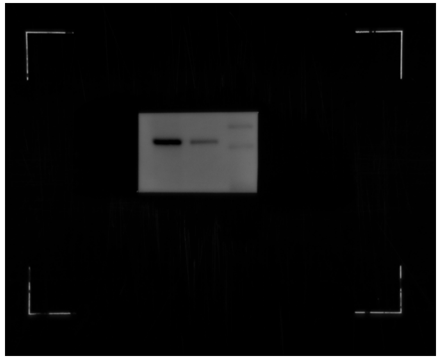

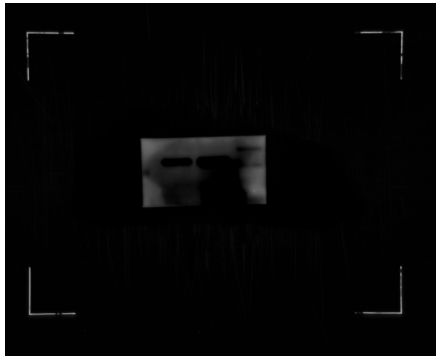

Tubulin

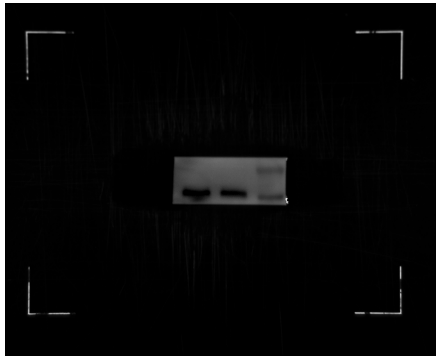

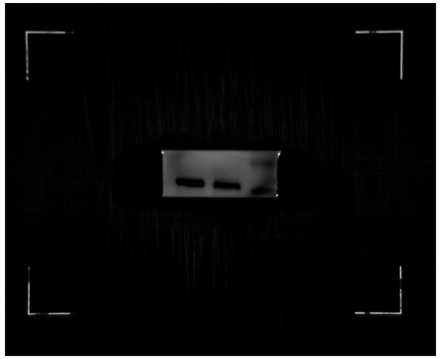

Fig.6E

Myc

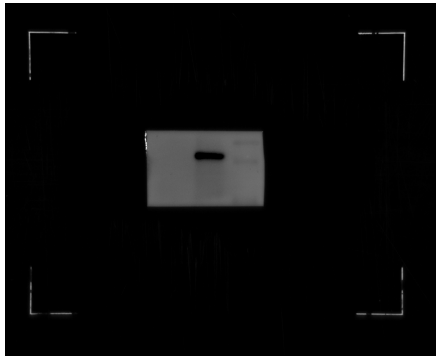

HA

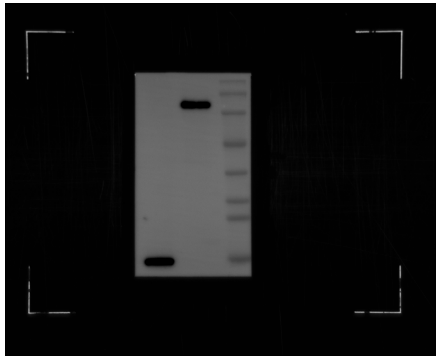

Myc

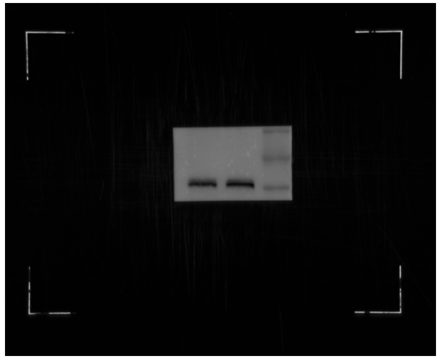

Fig.6F

TRIM21

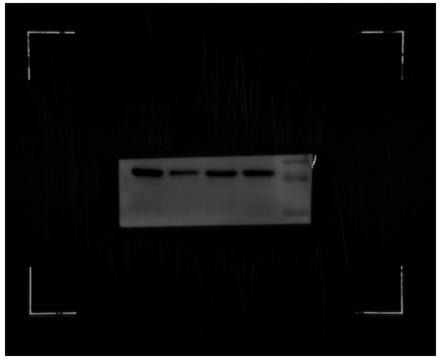

STAT1

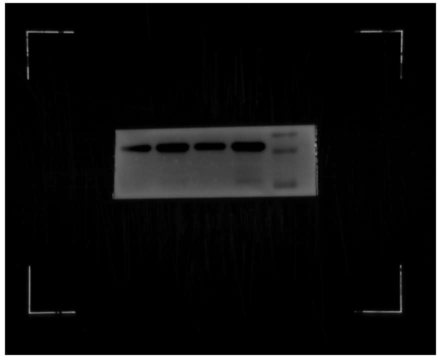

Tubulin

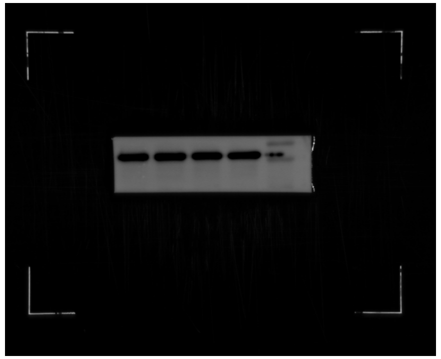

Fig.6G

HA

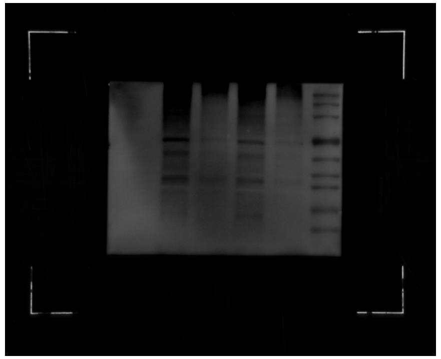

STAT1

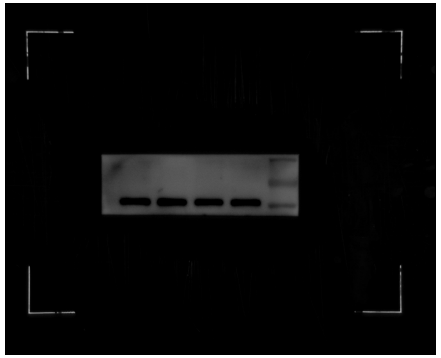

Fig.6H

Ub

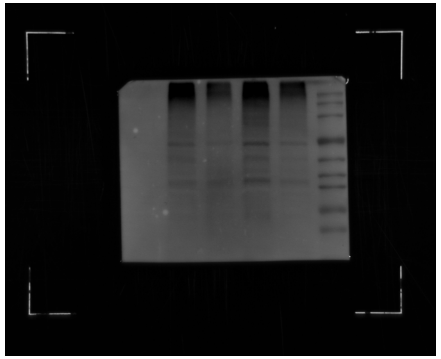

Tubulin

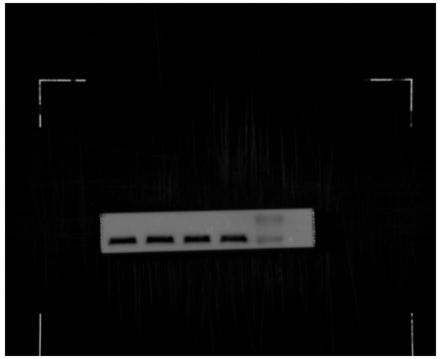

TRIM21

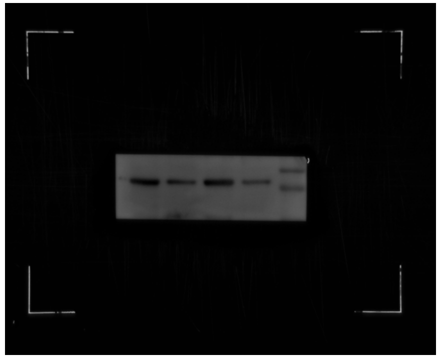

TREM2

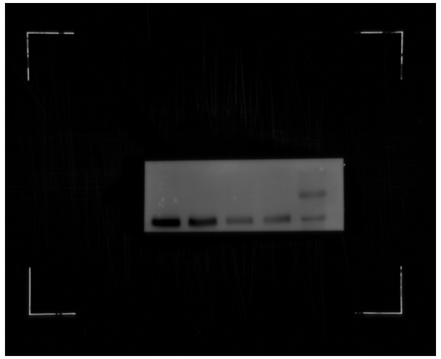

Fig.6I

Myc

TREM2

STAT1

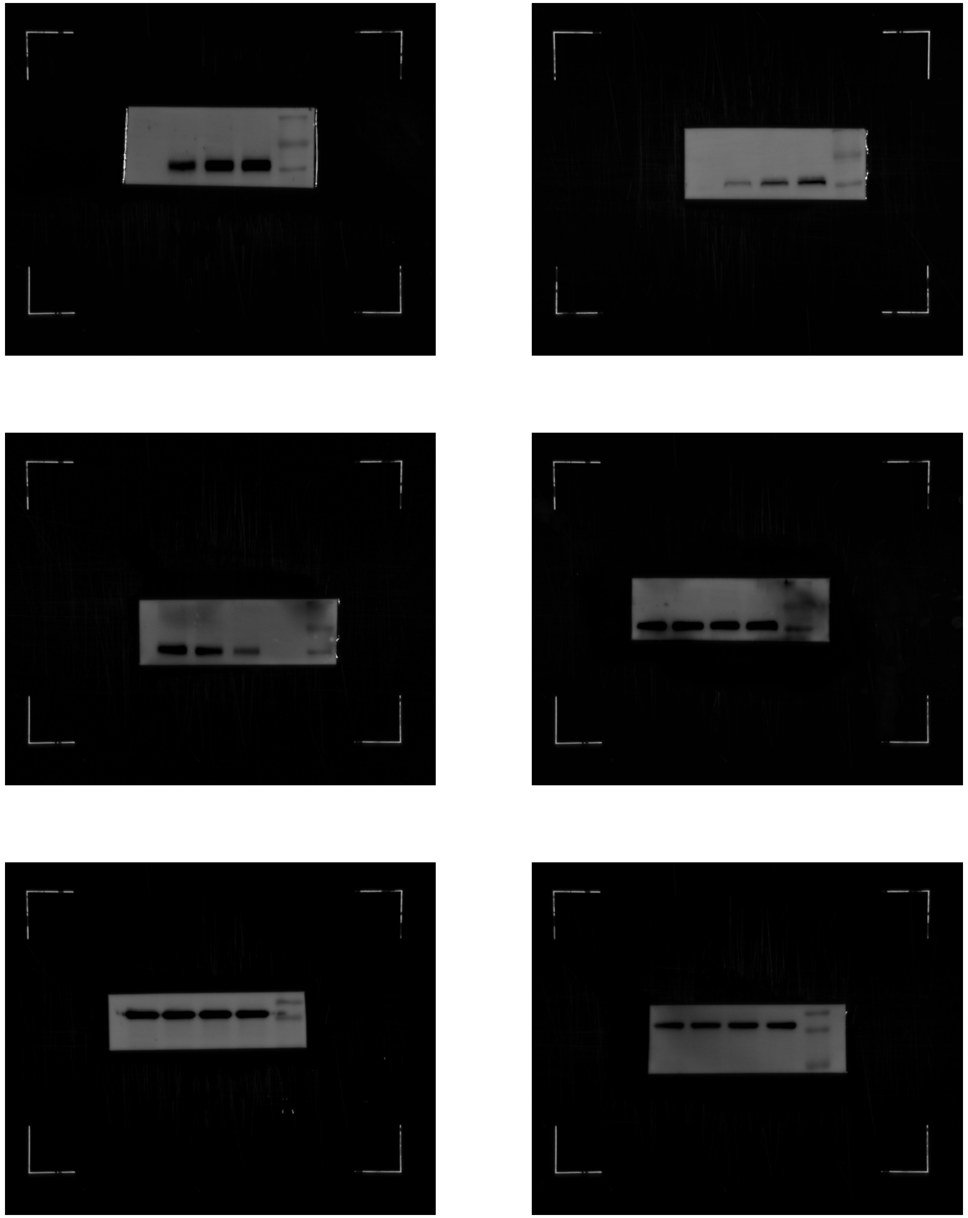

Fig.6J

STAT1

Flag

Myc

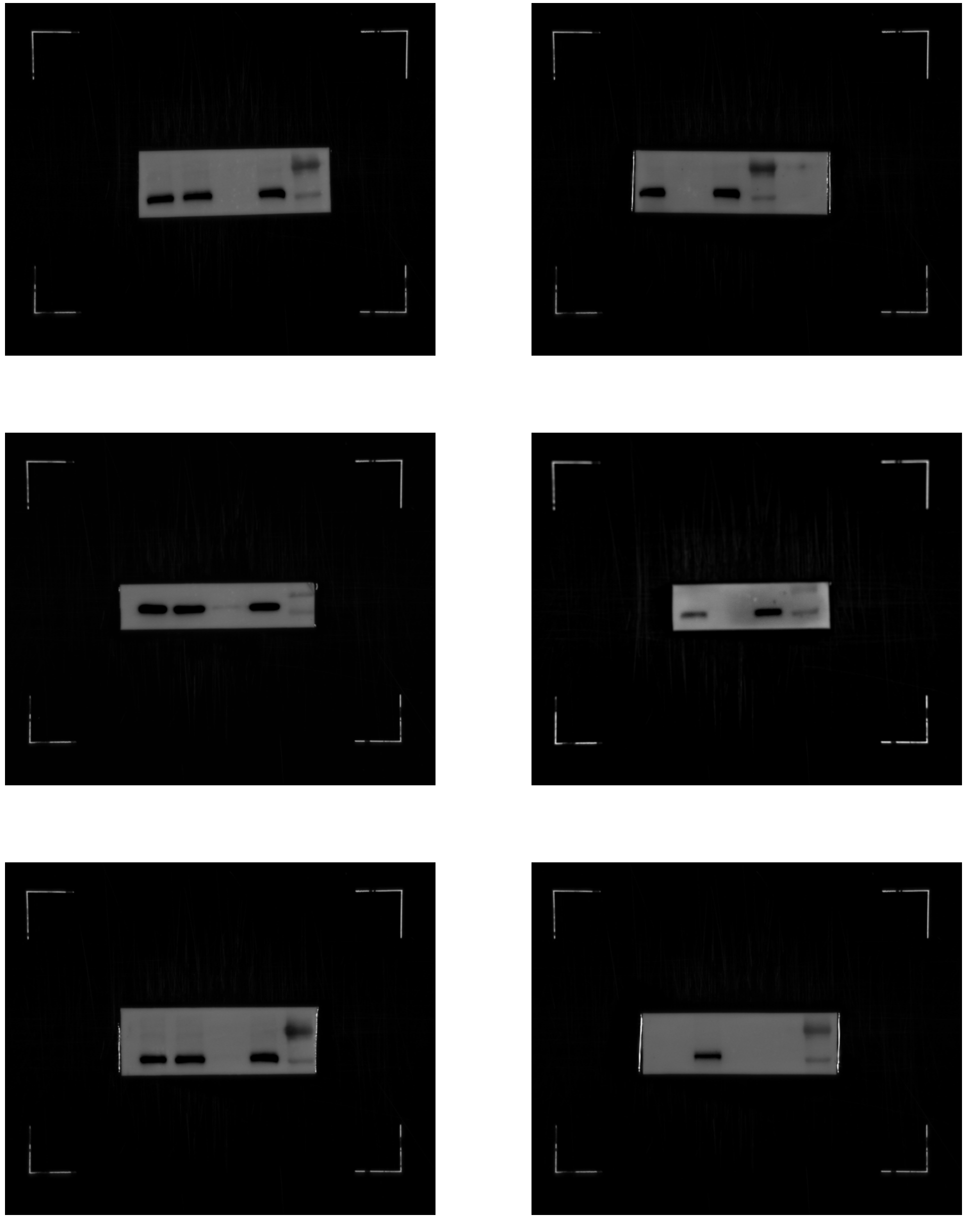

Fig.6K

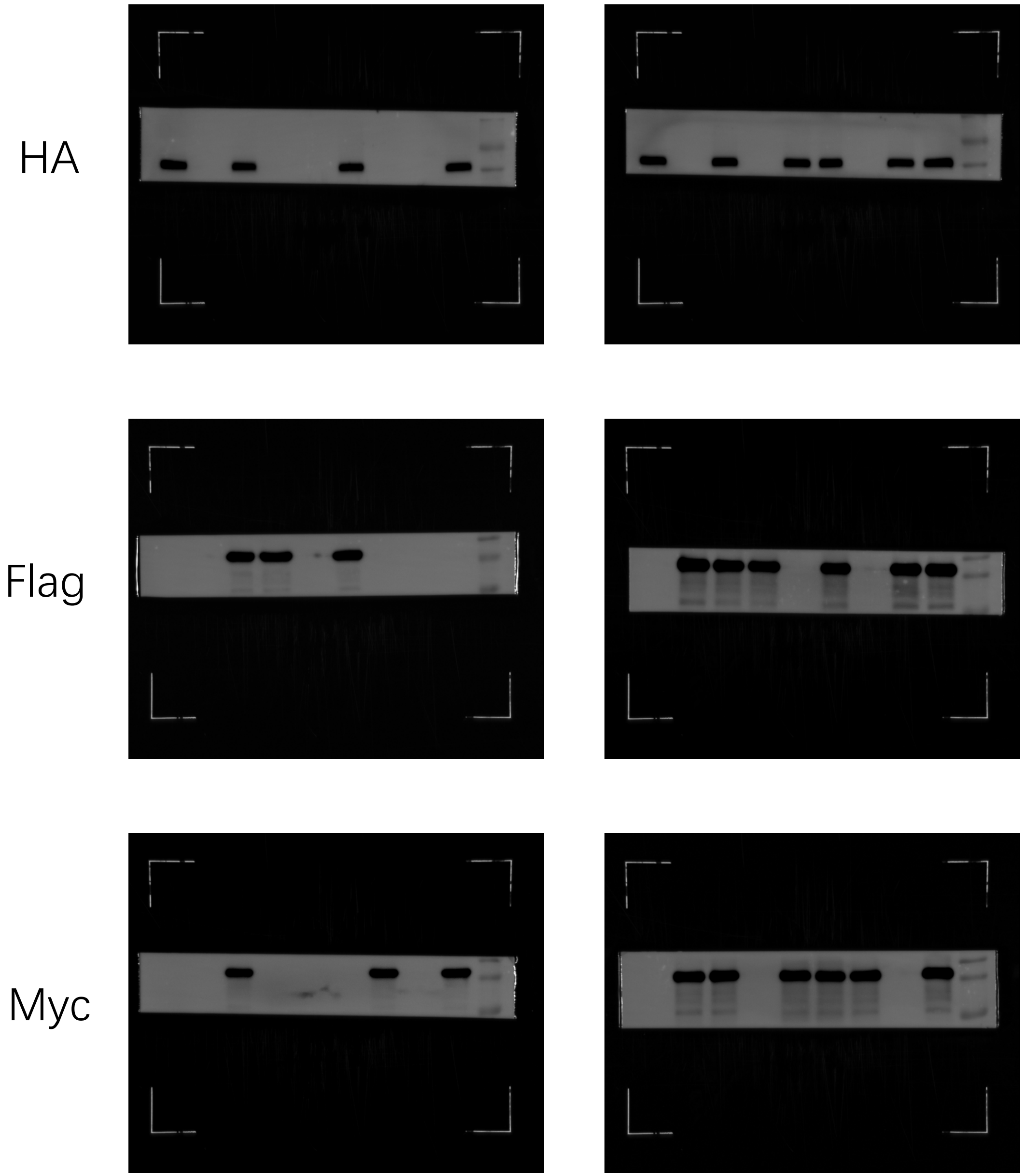

Fig.6L

TREM2

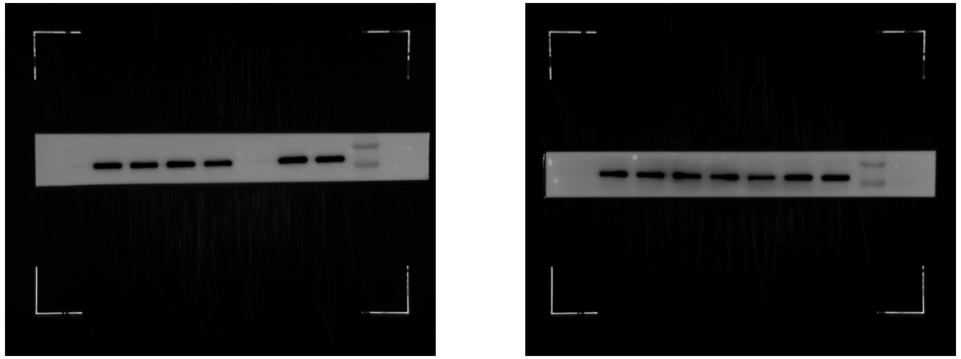

Tubulin

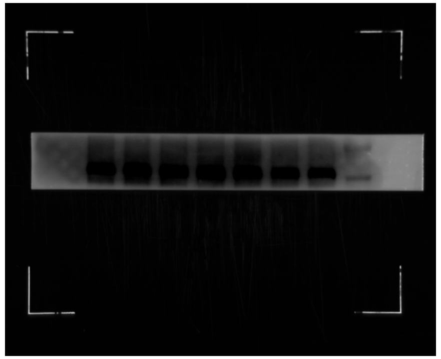

TRIM21

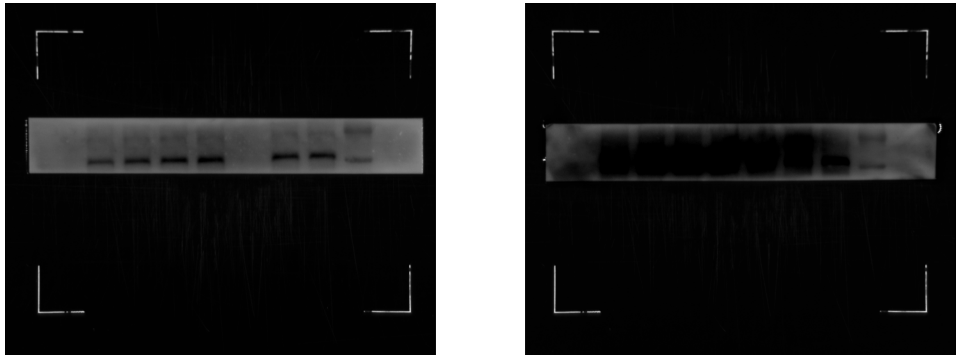

STAT1

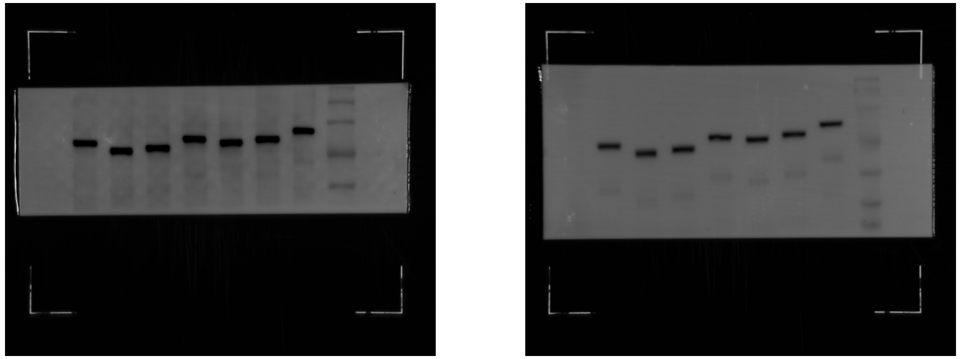

Fig.7A

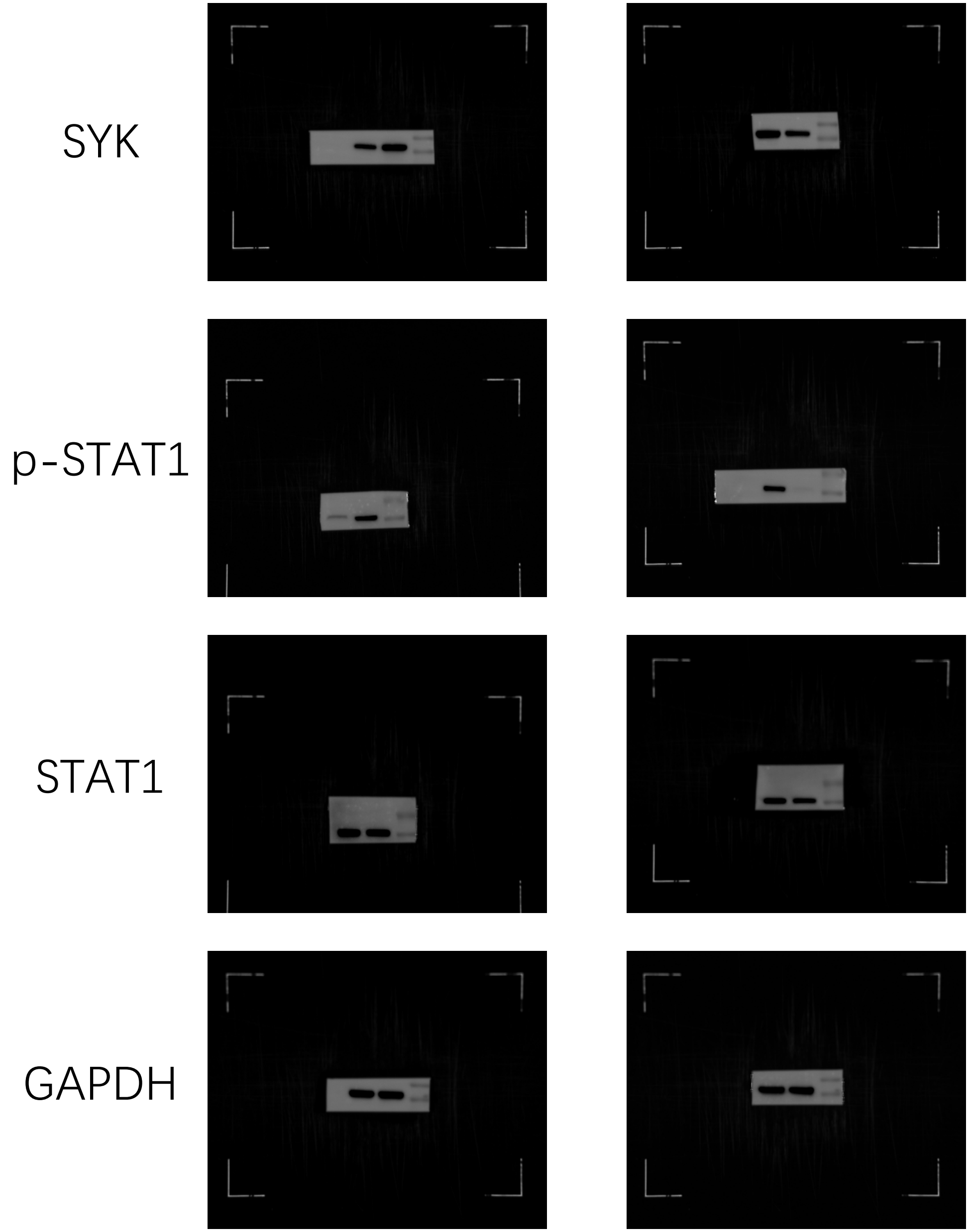

Fig.7B

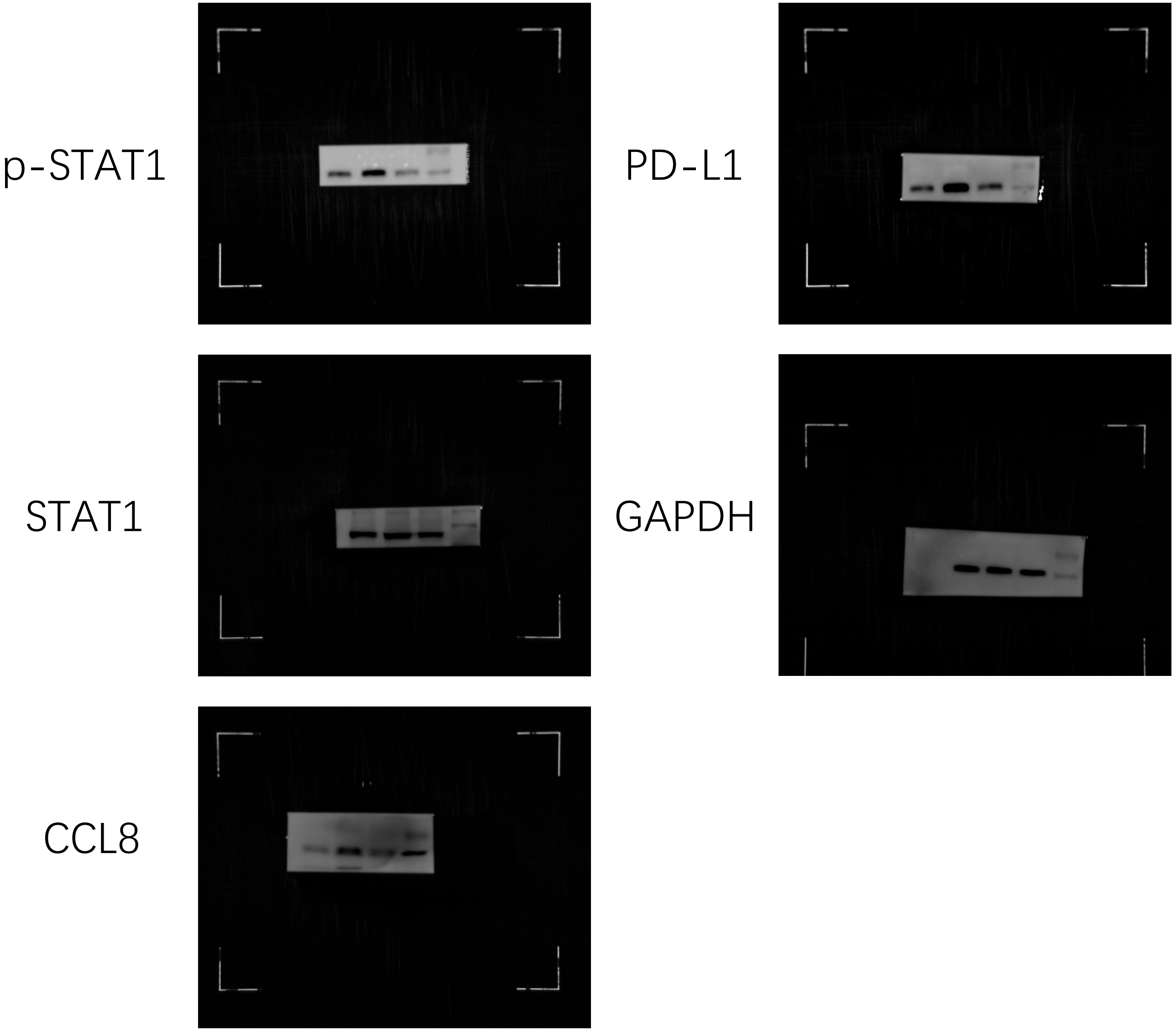

Fig.7C

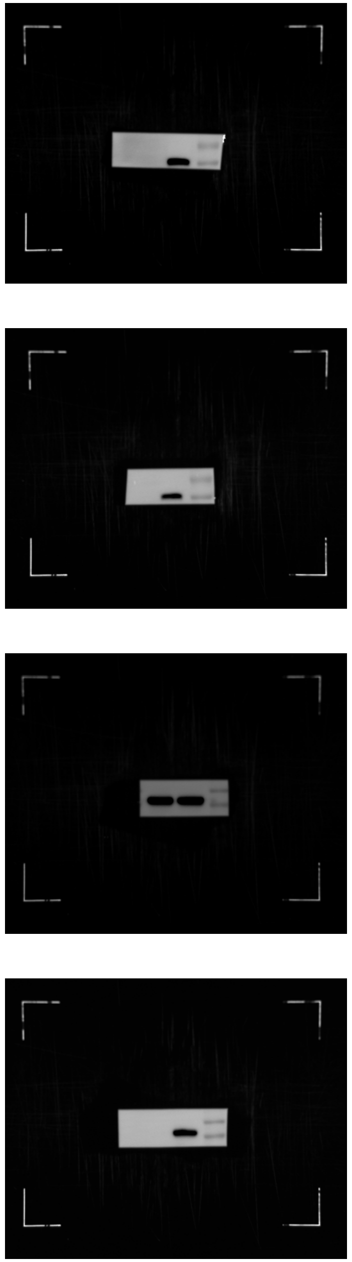

Fig.7D

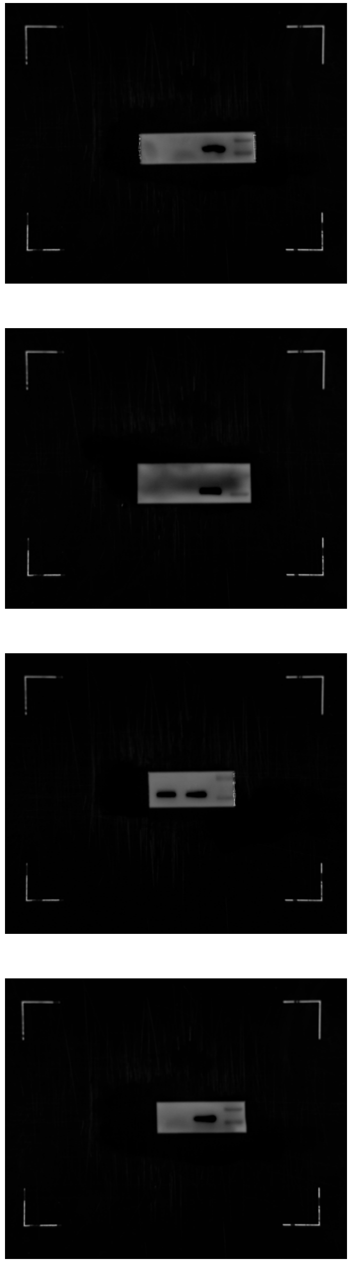

Fig.7E

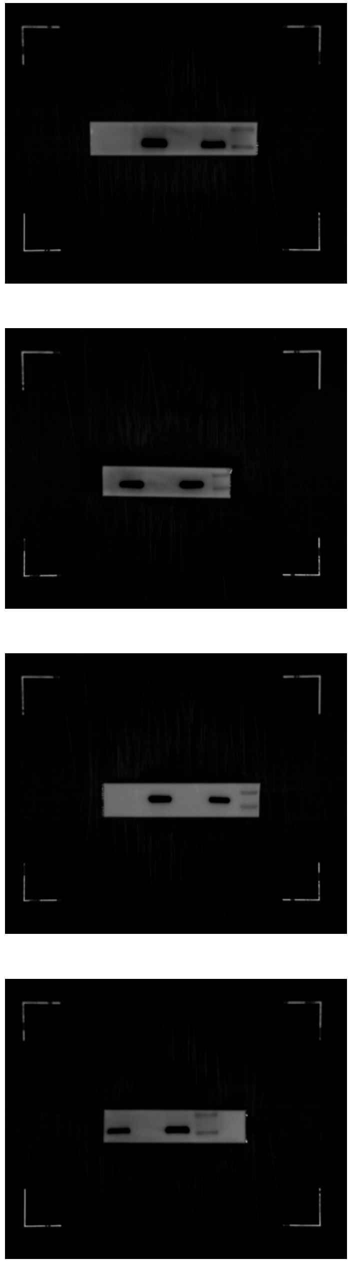

Fig.7F

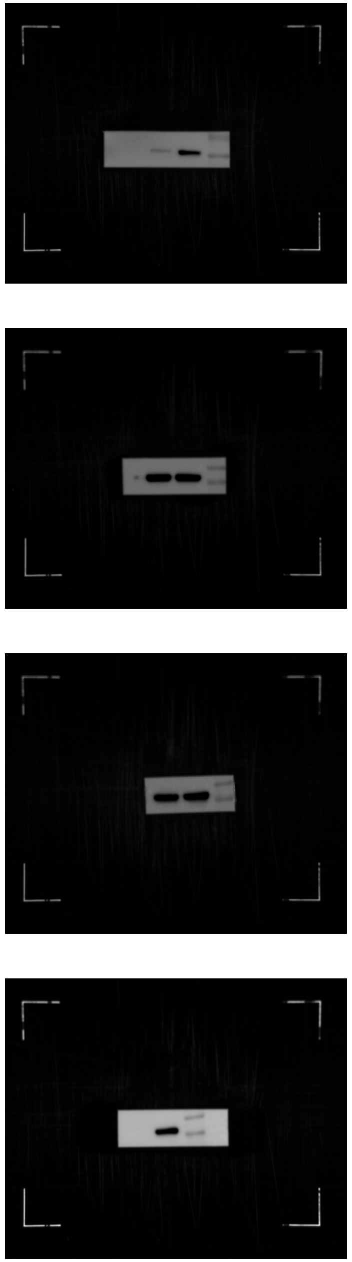

Fig.7G

Flag
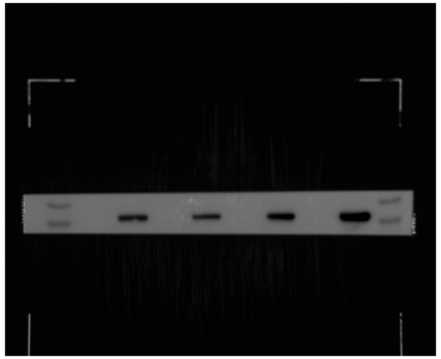

HA
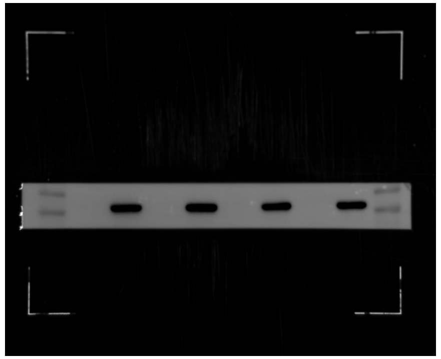

HA
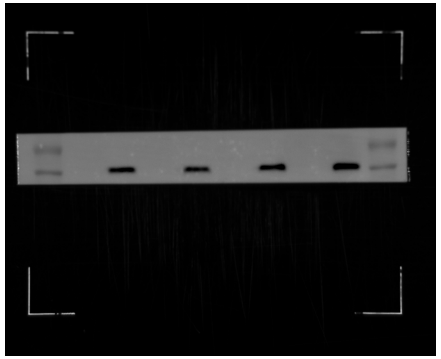

TREM2
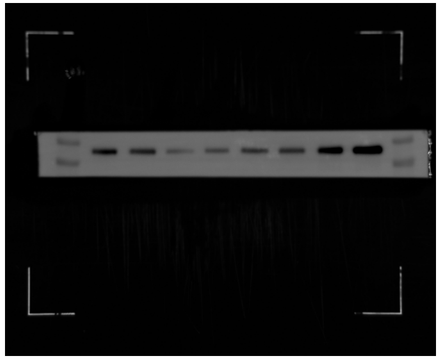

Flag
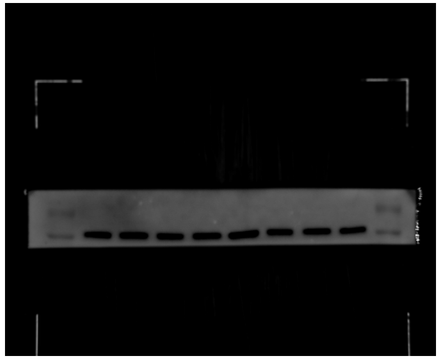

Tubulin
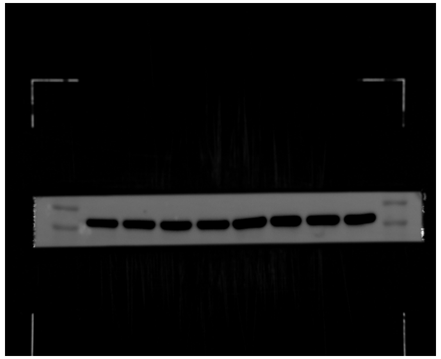

Fig.7H

His
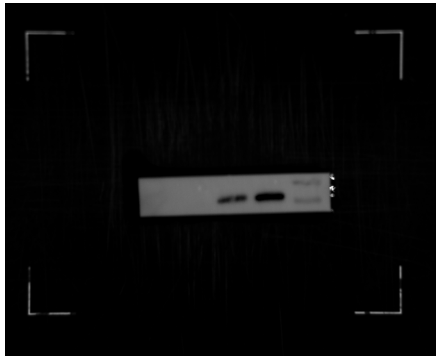

Flag
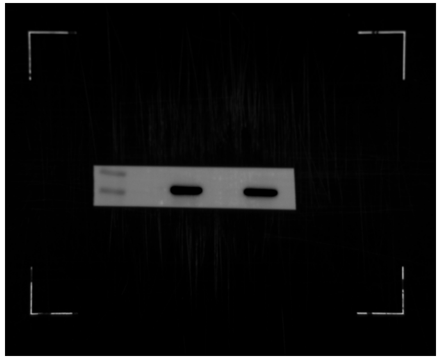

HA
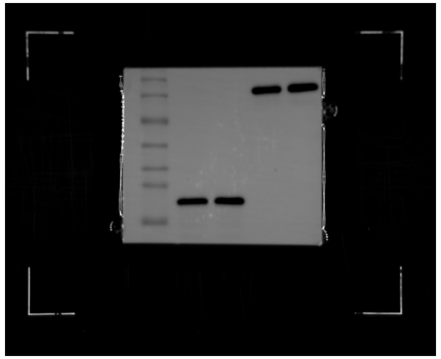

His
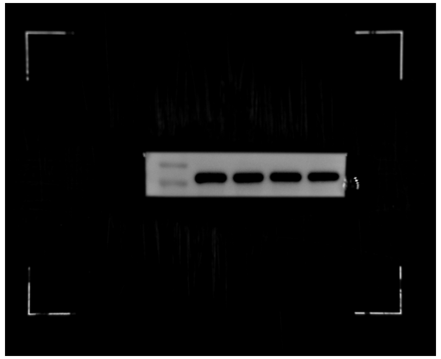

Fig.7I

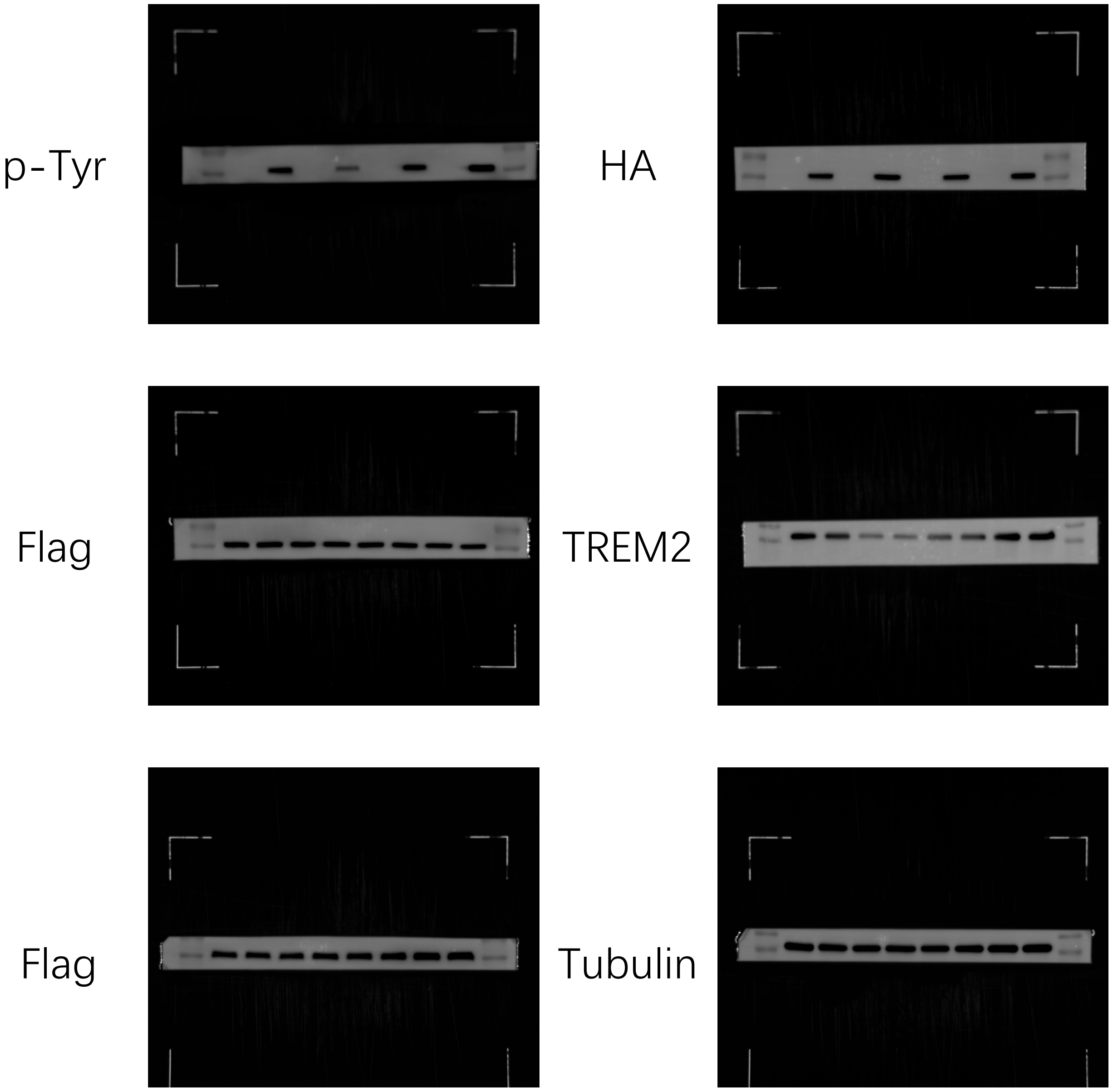

Fig.7J

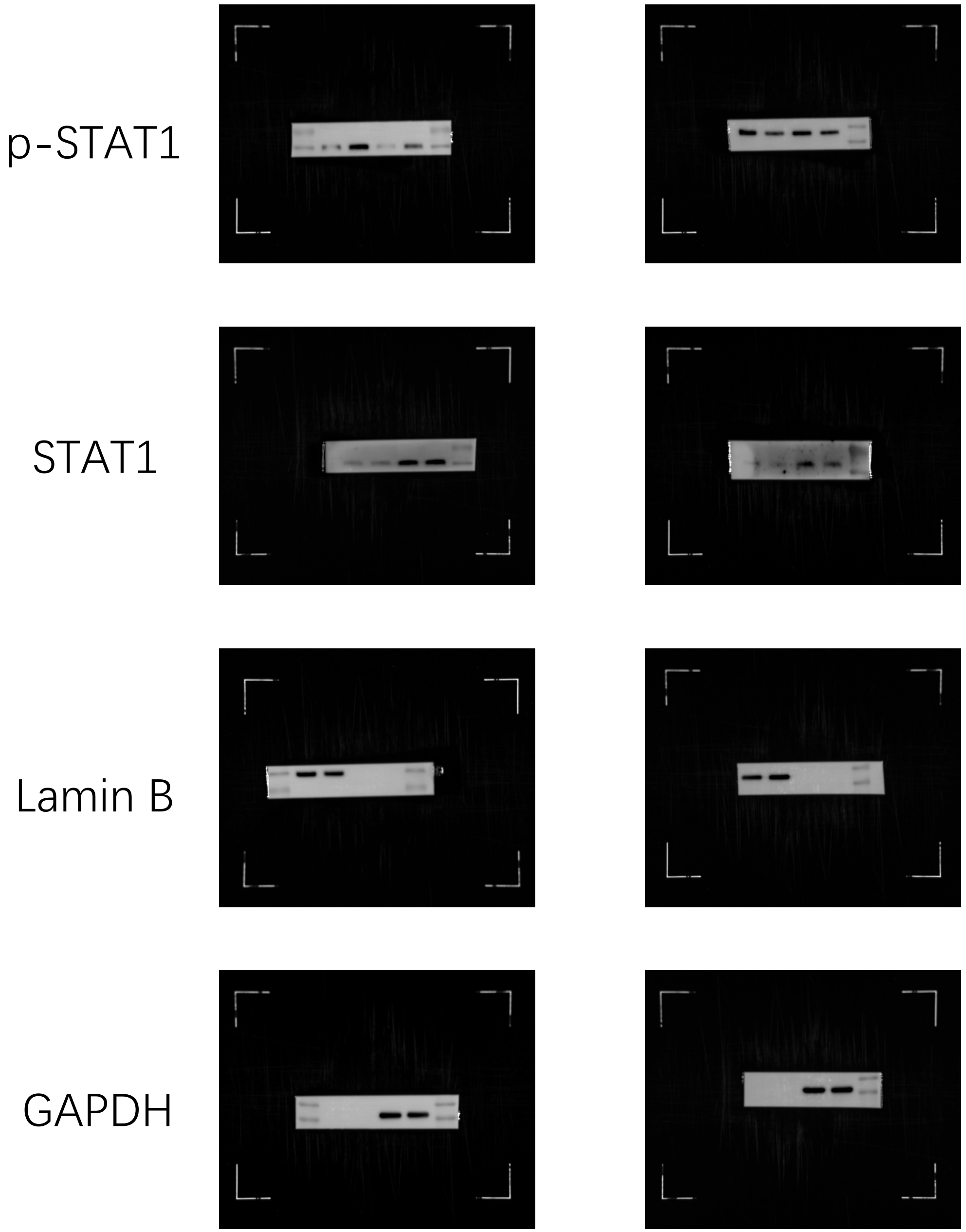

Fig.S7

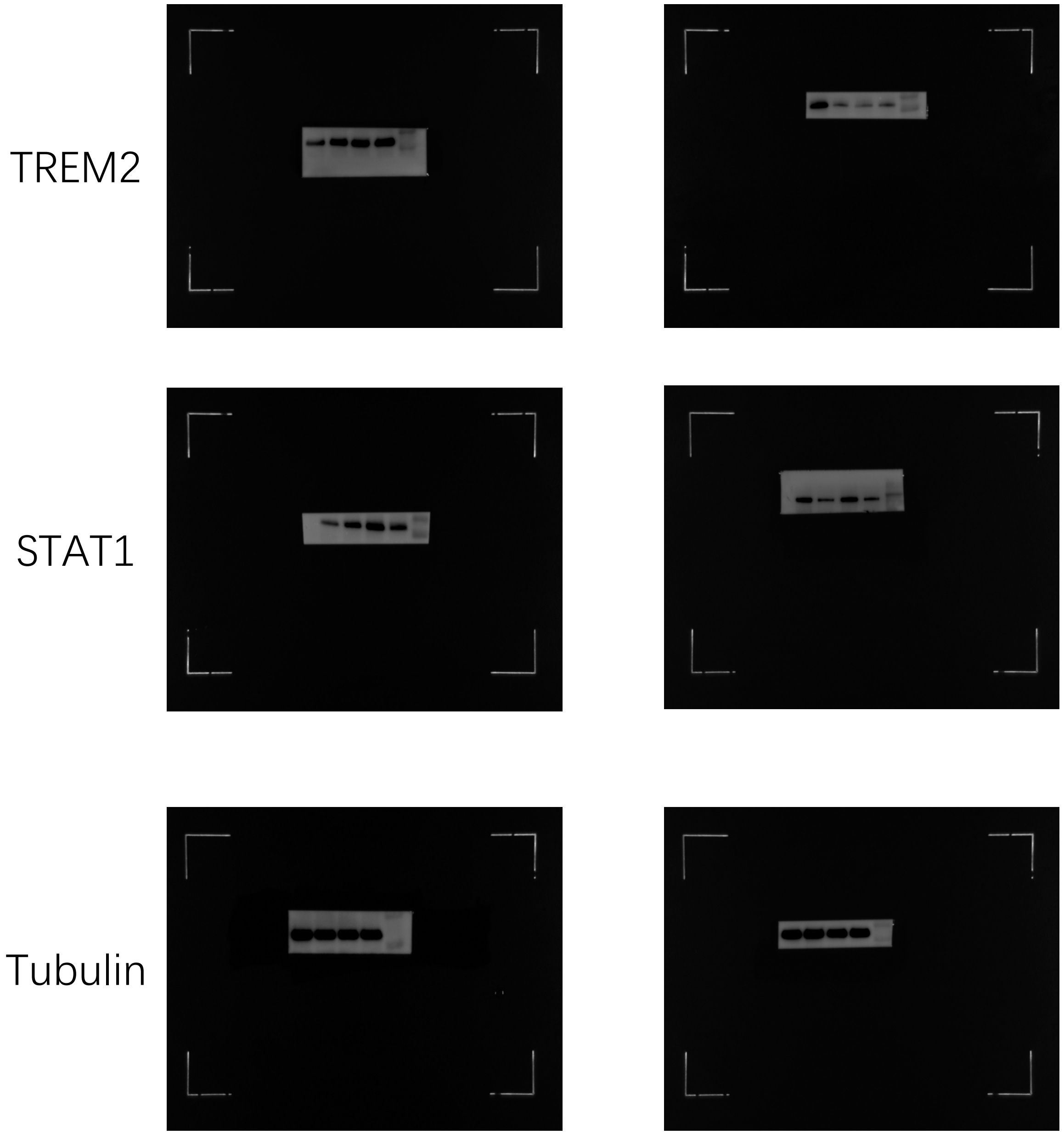

Supplement: Supplementary file 3 — Supplemental Material [file 41419_2025_8198_MOESM3_ESM.pdf]
